# A glycoprotein B-neutralizing antibody structure at 2.8 Å uncovers a critical domain for herpesvirus fusion initiation

**DOI:** 10.1038/s41467-020-17911-0

**Published:** 2020-08-18

**Authors:** Stefan L. Oliver, Yi Xing, Dong-Hua Chen, Soung Hun Roh, Grigore D. Pintilie, David A. Bushnell, Marvin H. Sommer, Edward Yang, Andrea Carfi, Wah Chiu, Ann M. Arvin

**Affiliations:** 1 Department of Pediatrics, Stanford University School of Medicine, Stanford, CA 94305 USA; 2 GSK Vaccines, Cambridge, MA 02139 USA; 3 Structural Biology, Stanford University School of Medicine, Stanford, CA 94305 USA; 4 Department of Biological Sciences, Institute of Molecular Biology & Genetics, Seoul National University, Seoul, 08826 Korea; 5 Bioengineering, Stanford University School of Medicine, Stanford, CA 94305 USA; 6 Microbiology and Immunology, Stanford University School of Medicine, Stanford, CA 94305 USA; 7 Division of Cryo-EM and Bioimaging SSRL, SLAC National Accelerator Laboratory, Menlo Park, CA 94025 USA; 8 Present Address: Division of Cryo-EM and Bioimaging SSRL, SLAC National Accelerator Laboratory, Menlo Park, CA 94025 USA; 9 Present Address: Division of Cryo-EM and Bioimaging SSRL, SLAC National Accelerator Laboratory, Menlo Park, CA 94025 USA

**Keywords:** Antibodies, Herpes virus, Viral membrane fusion, Virus-host interactions, Cryoelectron microscopy

## Abstract

Members of the *Herpesviridae*, including the medically important alphaherpesvirus varicella-zoster virus (VZV), induce fusion of the virion envelope with cell membranes during entry, and between cells to form polykaryocytes in infected tissues. The conserved glycoproteins, gB, gH and gL, are the core functional proteins of the herpesvirus fusion complex. gB serves as the primary fusogen via its fusion loops, but functions for the remaining gB domains remain unexplained. As a pathway for biological discovery of domain function, our approach used structure-based analysis of the viral fusogen together with a neutralizing antibody. We report here a 2.8 Å cryogenic-electron microscopy structure of native gB recovered from VZV-infected cells, in complex with a human monoclonal antibody, 93k. This high-resolution structure guided targeted mutagenesis at the gB-93k interface, providing compelling evidence that a domain spatially distant from the gB fusion loops is critical for herpesvirus fusion, revealing a potential new target for antiviral therapies.

## Abstract (web summary)

Herpesvirus virions have an outer lipid membrane dotted with glycoproteins that enable fusion with cell membranes to initiate entry and establish infection. Here the authors elucidate the structural mechanism of a neutralizing antibody derived from a patient infected by the herpesvirus varicella-zoster virus and targeted to its fusogen, glycoprotein-B.

## Introduction

Members of the *Herpesviridae* are pathogens of humans and animals that cause a wide range of medically and economically important diseases^1^. Herpesvirus virions have an outer lipid membrane studded with glycoproteins that enable fusion with cell membranes to initiate entry and establish infection. The most conserved herpesvirus protein, glycoprotein B (gB), is classified as a type III fusogen due to its structural similarities with vesicular stomatitis virus (VSV) G protein and baculovirus gp64^2–8^. In contrast to these fusogens, gB requires additional virally encoded glycoproteins, gH and gL, which are implicated in membrane binding and priming of the gB trimer, to induce membrane fusion^9^. Together, gB/gH-gL form the core herpesvirus fusion complex. Herpesvirus gB has been predicted to transition from a prefusion to a postfusion state^2–7^, but the molecular dynamics of the fusion complex is not understood.

Structures of the gB ectodomains of herpes simplex virus 1 (HSV-1), HSV-2, pseudorabies virus (PRV), human cytomegalovirus (HCMV), and Epstein Barr virus (EBV), determined by X-ray crystallography, have identified five domains (I to V) that are similar to those of VSV G protein^2–6,10,11^. The C-terminal domain (CTD) structure of the HSV-1 gB was obtained by X-ray crystallography at 3.6 Å resolution^12^. However, only low-resolution structures (>24 Å) of herpesvirus gB in a putative prefusion form have been identified on exosomes derived from HSV-1 gB transfected cells and on HCMV particles using cryogenic electron tomography (cryo-ET)^10,13^. This has limited the ability to accurately model prefusion conformations of gB.

Monoclonal antibodies (mAbs) to herpesvirus gB orthologues that neutralize viral infection are important for mapping functional domains because their activity depends on binding to gB before membrane fusion^4,14–19^. Antibodies that bind to DI, DII, and DIV of gB orthologues have neutralizing activity against several herpesviruses^4,15,20–23^. Although the molecular interactions for some of these antibodies with gB residues have been defined, it is currently not known whether these gB residues have roles in fusion function or virus infection.

Varicella-zoster virus (VZV) is a highly infectious, human host restricted alphaherpesvirus that causes varicella (chickenpox), establishes latency in sensory ganglion neurons and can reactivate to manifest as zoster (shingles)^24^. In addition to virion entry fusion, cell–cell fusion (abbreviated as cell fusion) is fundamental for VZV pathogenesis. Characteristic polykaryocytes form within tissues in vivo and are modeled in vitro by syncytia formation during MeWo cell infection^25,26^. Critically, adverse health effects are directly linked to the capability of VZV to overcome the usual constraint against fusion between differentiated host cells, causing fusion of ganglion neurons and satellite cells associated with postherpetic neuralgia (PHN), and fusion of vascular endothelial cells (giant cell arteritis) linked to strokes^27–29^.

VZV gB, a 931 amino acid (aa) protein encoded by open reading frame (ORF) 31, together with the VZV gH-gL heterodimer, trigger cell fusion in vitro, in the absence of other viral proteins^30–32^. The purpose of this study was to use the neutralizing human mAb 93k as a pathway to biological discovery of gB functional domains. A 2.8-Å resolution cryo-EM structure of native, full-length VZV gB in complex with mAb 93k Fab fragments was determined, revealing residues within gB DIV that were then shown to be essential for membrane fusion by evaluating DIV mutants in a virus-free assay. Mutagenesis of the VZV genome demonstrated their significance for gB fusion functions necessary to produce infectious extracellular VZV virions and for cell fusion to form syncytia. These findings have implications for modeling the transition of gB from prefusion to postfusion conformations. This study is highly relevant for developing novel therapies that inhibit infection by disrupting gB DIV-dependent molecular mechanisms of cell entry or cell fusion by members of the *Herpesviridae*.

## Results

### Human mAb 93k neutralizes VZV and inhibits fusion

**Fig. 1:**
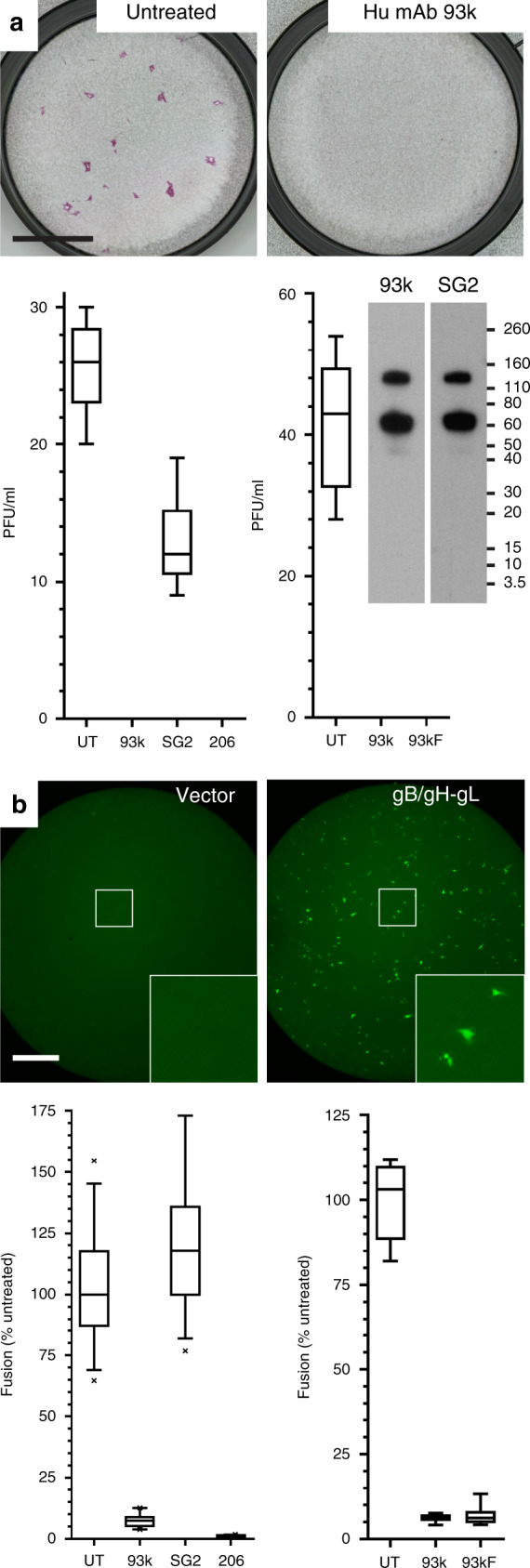
Human mAb 93k neutralizes VZV by fusion inhibition. **a** Cell-free VZV plaque neutralization assay. Immunohistochemical staining of VZV-infected MeWo cells untreated or treated with mAb 93k. Scale bar (black) 5 mm. The box and whisker plots (5–95 percentile) of VZV neutralization with mAbs 93k, SG2 (murine anti-gB) and 206 (murine anti-gH), or 93k Fab fragments (93kF) represent *n* = 6 samples examined over two independent experiments. Inset – western blots of purified, full-length VZV gB resolved by SDS–PAGE and detected using the mAbs 93k or SG2. Numbers represent molecular weights (kDa) for the protein standard. VZV gB is cleaved by furin^33^; both uncleaved (130 kDa) and cleaved (65 kDa) gB is detected by mAbs 93k and SG2. **b** Cell–cell fusion inhibition assay. Fluorescence microscopy of fused cells (GFP; green) generated by co-transfection of gB/gH-gL in the stable reporter fusion assay (SRFA). Scale bar (white) 1 mm. The box and whisker plots (5–95%) of fusion inhibition with mAbs 93k, SG2 and 206 or the 93kF represent *n* = 34 (mAbs) or *n* = 8 (Fab) samples examined over two independent experiments. Source data are provided as a Source Data file.

The biological activity of mAb 93k or its Fab fragments was demonstrated by neutralization of a cell-free inoculum of the VZV strain, pOka (Fig. 1a). Importantly, 93k also reduced gB/gH-gL-mediated membrane fusion by 95% in the stable reporter fusion assay (SRFA) in the absence of other viral proteins (Fig. 1b). Similar to mAb 93k, the anti gB mAb SG2^33^ bound to full-length gB that was purified from infected MeWo cells (Fig. 1a Inset; Supplementary Fig. 2b) but had limited neutralizing activity and failed to inhibit fusion (Fig. 1a, b). The neutralization of cell-free VZV and the fusion inhibition properties of mAb 93k and its Fab fragment confirmed that the 93k epitope was exposed in a prefusion conformation of gB, indicating that mAb 93k could be used to identify residues involved in fusion initiation.

### Near atomic resolution structure of the gB-93k interface

**Fig. 2:**
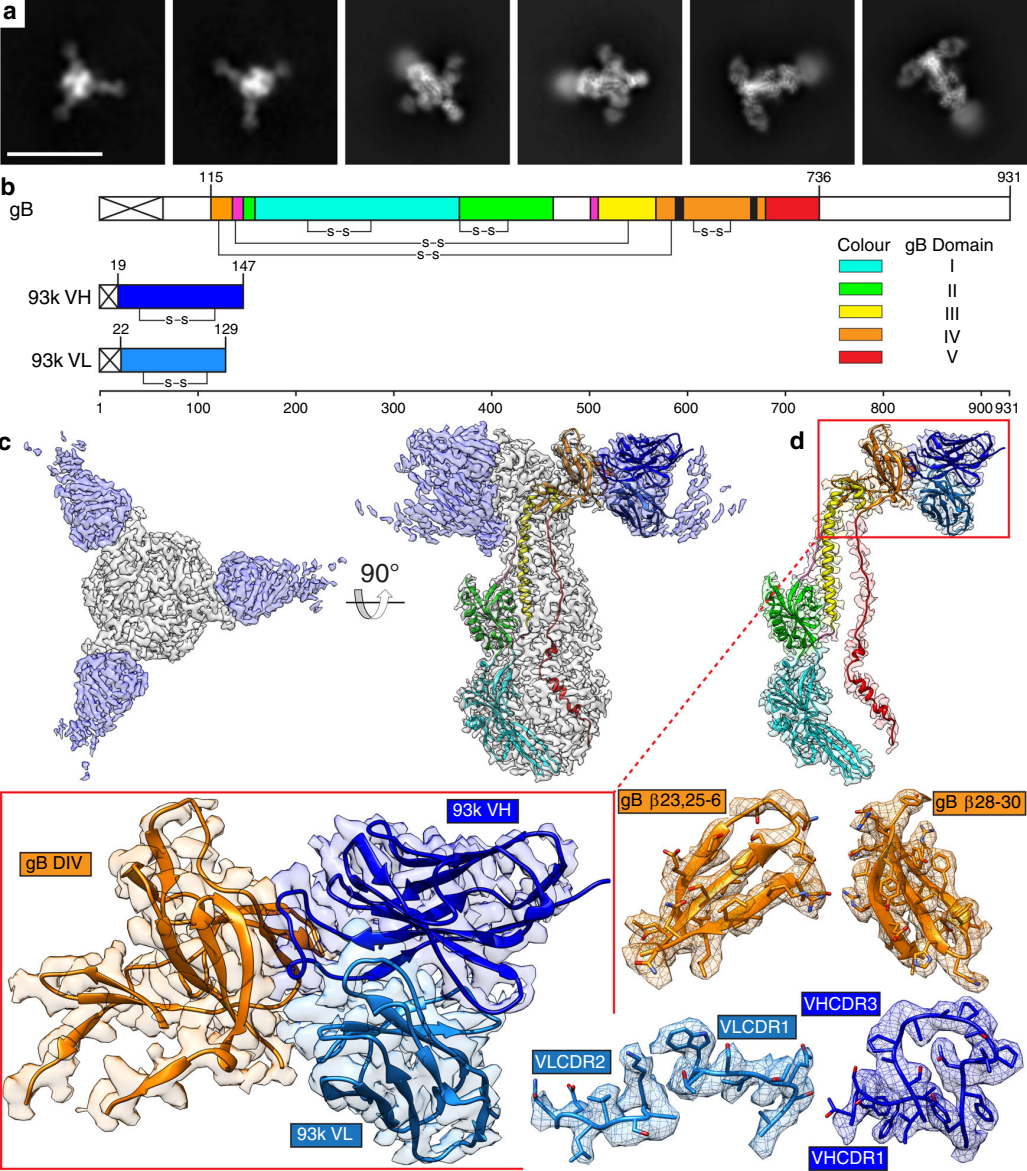
Near atomic resolution cryo-EM structure of human neutralizing mAb 93k Fab fragments bound to native VZV gB. **a** Representative 2D class averages used to generate the 2.8-Å cryo-EM structure of the gB-93k complex. Scale bar (white) 20 nm. **b** Linear maps of VZV gB, 93k VH and 93k VL, are drawn to scale and each domain colored accordingly: DI (cyan), DII (green), DIII (yellow), DIV (orange), DV (red), linker regions (hot pink), 93k VH (blue) and 93k VL (light blue). The predominant interaction sites of the 93k VHCDR3 loop at gB β23 and β30 are highligted in black on the gB linear structure. **c** Cryo-EM map of the VZV gB-93k complex. The gB trimer (gray) and the 93k Fab fragments (blue) are segmented (see Supplementary Movie 1). **d** Segmentation of the cryo-EM map for one VH and VL chain of a 93k Fab fragment bound to a protomer of VZV gB (See Supplementary Movie 1). The structures of VZV gB and 93k VH and VL are represented as ribbons. Expanded views of the cryo-EM map for gB DIV and the 93k VH and VL are highlighted by the red boxes with the structure represented by ribbons and the cryo-EM map desity shown. Segementation and amino acid sidechains are shown for the exploded view of gB DIV β-strands (β23, 25–26 and β28-30) and mAb 93k CDR loops (VHCDR1, VHCDR3, VLCDR1, and VLCDR2) that form the gB-93k interface (see Supplementary Movie 2).

To define the molecular interactions formed between native gB and mAb 93k, a 2.8-Å structure of the gB-93k Fab complex was generated by using single particle cryo-EM, coupled with stringent particle selection and validation of local resolution (ResMap^34^), and feature resolvability through Q-score assignment (MapQ^35^) to amino acid side chains (Fig. 2a–d; Supplementary Figs. 1–3; Supplementary Tables 1–4; Supplementary Movie 1 and 2). Since gB was purified from VZV-infected MeWo cells, it was anticipated that alternative conformations of gB would be isolated, including prefusion forms. However, class averages were not identified in the cryo-EM micrographs that resembled a prefusion form of gB in complex with mAb 93k Fab fragments, underscoring the highly metastable status of the prefusion form of herpesvirus gB. Local resolution of the cryo-EM map ranged from 2.3 to 4 Å with expected visibility of the amino acid side-chain densities for the gB ectodomain to ensure the assignment of sequence identity (Fig. 2c, d; Supplementary Figs. 2f and 3). The VZV gB ectodomain was composed of five domains, DI to DV, with 13 helices and 31 β-strands, similar to herpesvirus orthologue structures in their postfusion conformations (Fig. 2c, d; Supplementary Table 2). Each protomer was stabilized by five disulfide bonds, conserved among alpha-, beta-, and gammaherpesviruses (Fig. 2b, Supplementary Table 3). C122/C584 stabilized the non-contiguous regions of DIV (N-terminal, aa115-136 and C-terminal, aa570-681) and C139/C540 bridged the distal end of the long central helix of DIII (α8) with the linker to DII. C213/C277, C369/C417, and C608/C645 formed stabilizing intra-domain interactions within domains I, II, and IV respectively.

**Fig. 3:**
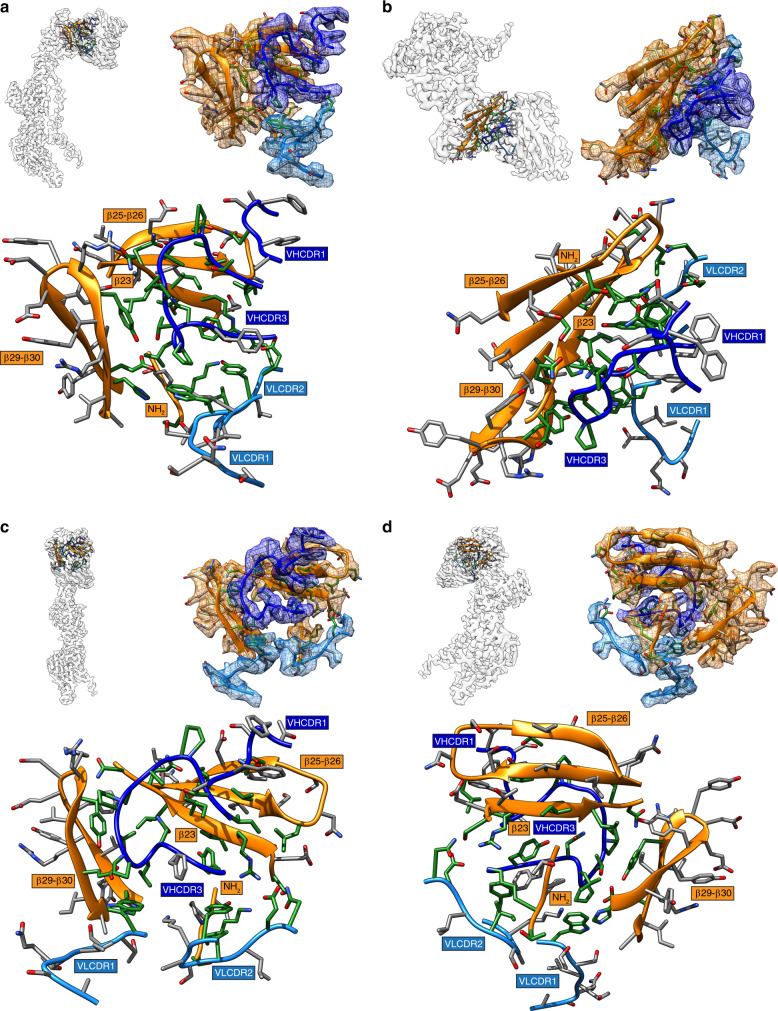
Human mAb 93k forms a stable interface with gB Domain IV. **a**–**d** Location in the 2.8-Å cryo-EM map (top left panels) of the extracted densities (top right panels; scenes captured from Supplementary Movie 3) for the gB protomer and the associated bound 93k Fab. The densities of gB DIV (orange), 93k VH chain (blue) and 93k VL chain (light blue) are highlighted. A ribbon diagram and side chains of the amino acids at the extracted densities are shown with those highlighted in green representing the interactions formed at the gB-93k interface. The bottom panels duplicate the regions in the top right panels but without the extracted cryo-EM map densities. The β23, β25-26, β29-30, and the NH_2_ terminus of gB are highlighted with orange boxes, and the VHCDR1, VHCDR3, VLCDR1, and VLCDR2 are highlighted by blue boxes; VH – dark blue, VL – light blue.

**Fig. 4:**
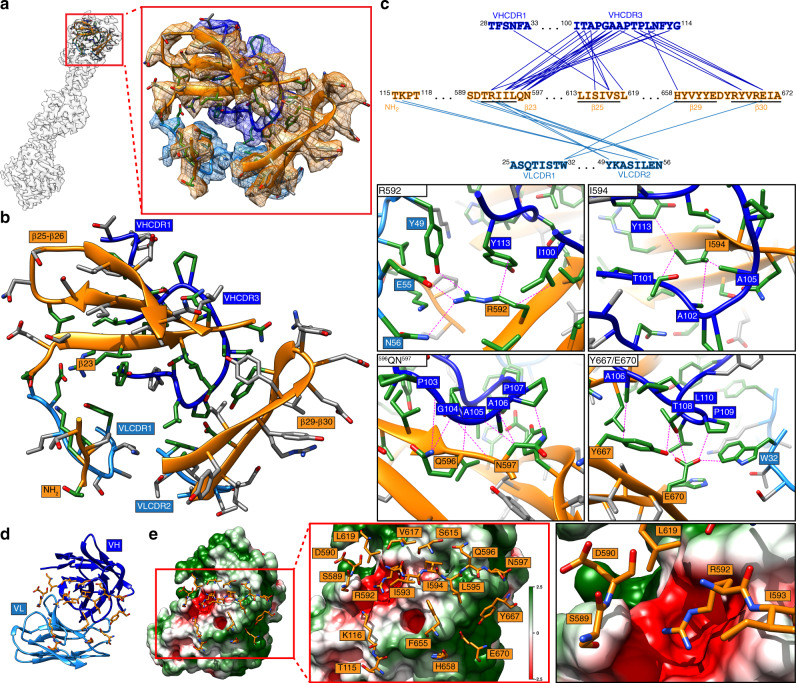
Molecular interactions at the VZV gB-93k interface. **a** Location in the 2.8-Å cryo-EM map and extracted densities of one gB protomer in complex with a 93k Fab fragment. The zoomed in panel shows the extracted densities at the gB-93k interface. **b**, **c** Representative scenes from Supplementary Movie 3. **b** Ribbon diagram of the amino acids from the extracted density at the gB-93k interface (**a**). Those highlighted in green represent interacting amino acids. The β23, β25-26, β29-30, and the NH_2_ terminus of gB are highlighted with orange boxes, and the VHCDR1, VHCDR3, VLCDR1, and VLCDR2 are highlighted by blue boxes; VH – dark blue, VL – light blue. **c** Molecular interactions between gB and the VH and VL chains of mAb 93k. The upper panel shows a linear map of the interactions (blue lines) between gB residues in β23, β25, β29, and β30 with those in VHCDR1, VHCDR3, VLCDR1, and VHCDR2 (not shown in Supplementary Movie 3). Underlined amino acids represent beta strands. The four lower panels show the interactions between gB residues R592, I594, ^596^QN^597^, and Y667/E670D with mAb 93k. Dotted lines (magenta) represent molecular interactions calculated using Find Contacts (Chimera, Supplementary Data 1). All interactions are shown in Supplementary Movie 3. **d**, **e** Orientations are equivalent to B. **d** The mAb 93k VH (blue) and VL (light blue) chains with gB interacting amino acid side chains (orange). **e** Surface electrostatic potential of the mAb 93k Fab VH and VL chains (calculated using APBS; Adaptive Poisson-Boltzmann Solver^69^) and zoomed in (middle and right panels) to highlight the gB interface residues represented in stick format (orange), which are scenes from Supplementary Movie 4. The potentials are on a red–white–green color map (−2.5 to 2.5) in units of kJ/mol/e.

The 2.8-Å cryo-EM map provided near atomic level details of the mAb 93k footprint on VZV gB identifying interactions between the variable heavy chain complementarity determining region 3 (VHCDR3) loop and regions in DIV, encompassing residues 589–597 of β23, 613–628 of β25-β26, and 658–670 of β28-30 (Figs. 2d, 3, and 4; Supplementary Fig. 4; Supplementary Spreadsheet 1). On one side of VHCDR3, sidechains of I100, A102, A105, and Y113 formed a hydrophobic network with gB residues R592 and I594 of β23, and V617 and L619 of β25 (Figs. 3 and 4; Supplementary Movie 3; Supplementary Table 4). The sidechain of gB R592 formed a cation-π interaction with the aromatic ring of VHCDR3 Y113 and fit into a negatively charged pocket within the 93k antigen binding site (Fig. 4b–d; Supplementary Movie 3 and 4). In addition, the carbonyl oxygen and backbone nitrogen of gB I593 and L595 formed H-bonding with the OH group of VHCDR3 Y113 and the sidechain of N111 respectively (Supplementary Movie 3). At the periphery of gB β23 and 93k interface, the sidechain of the gB Q596 and the backbone nitrogen of N597 H-bonded with the carbonyl oxygen of VHCDR3 residues P103 and G104, respectively, while the backbone nitrogen of VHCDR3 A106 H-bonded with the gB L595 carbonyl oxygen (Fig. 4c; Supplementary Movie 3). This marked a turning point of the interface after which residues of 93k interacted with β28-30, dominated by hydrophobic and Van der Waals contacts. The H-bond between gB E670 OE1 and VHCDR3 T108 OG1 was surrounded by hydrophobic interactions formed by gB β28-30 residues F655, H658, V660, and Y667, residues P107, P109, and L110 of VHCDR3, and W32 of the variable light chain CDR1 (VLCDR1; Fig. 4c; Supplementary Movie 3). Additional H-bonds and salt bridges peripheral to the gB-93k β-sheet interactions were formed between sidechain atoms of gB T624 and VHCDR1 N31, gB R592 and VLCDR2 Y49, and gB S589 and VLCDR2 N56. Thus, the 2.8-Å cryo-EM map revealed a complex network of hydrophobic and hydrophilic interactions at the gB-93k interface of postfusion gB with the strongest interactions between gB β23 and β30, and the 93k VHCDR3. Importantly, because mAb 93k has neutralizing activity through fusion inhibition (Fig. 1a, b), this suggested that residues within gB DIV β23 and β30 play a functional role in membrane fusion.

### Domain IV β23 residues are critical for gB fusion function

**Fig. 5:**
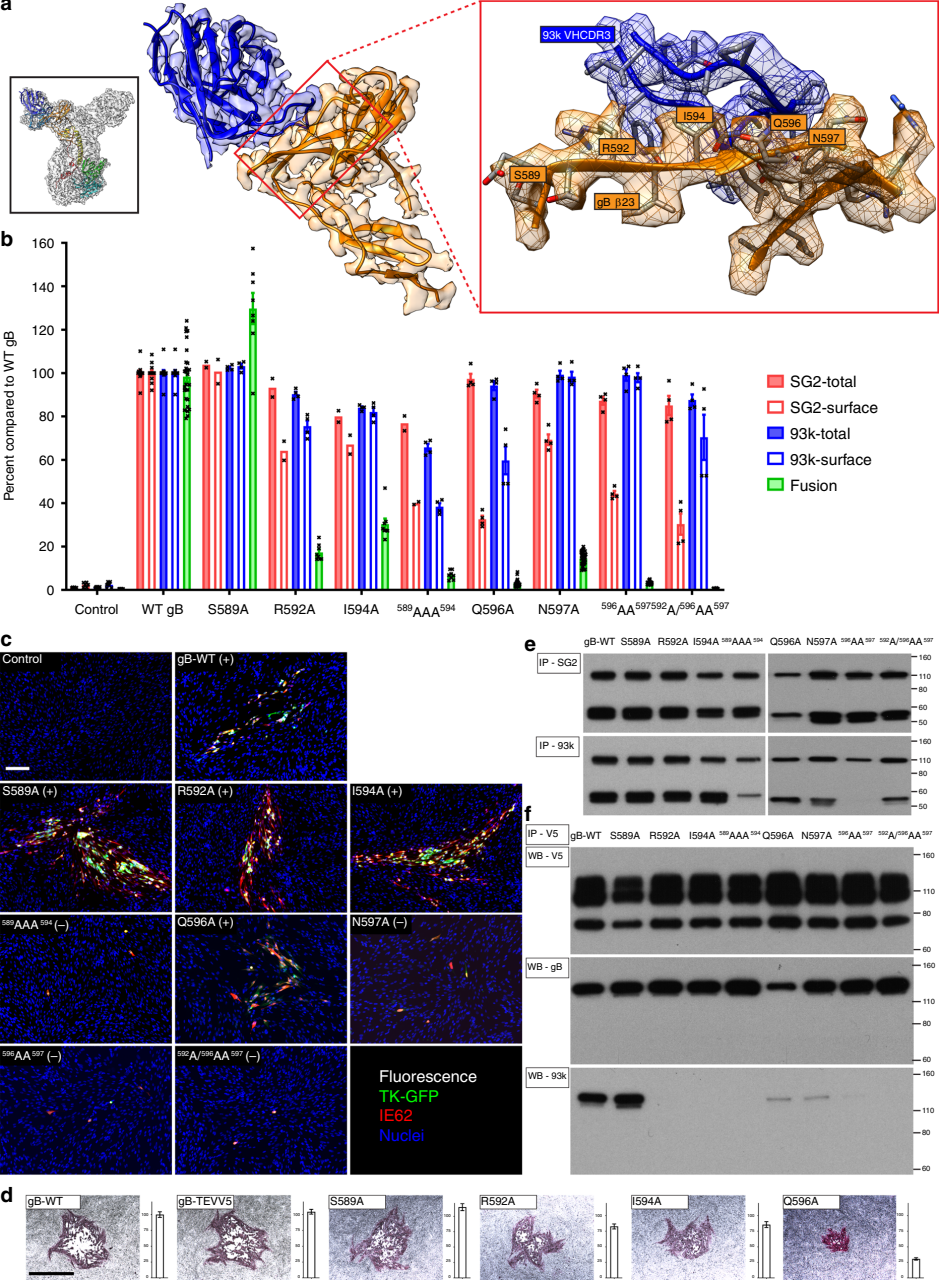
Domain IV β23 residues are critical for fusion function and VZV replication. **a** Near atomic structure of the mAb 93k binding site at VZV gB β23. The orientation of the complete gB-93k Fab map is shown in the small box in the top left-hand corner colored as for Fig. 1e. A portion of the cryo-EM map for gB domain IV chain A (Orange) and the bound 93k Fab (Blue) are shown. The red box on the right is a magnified view of β23 with the amino acid side chains on VZV gB shown and those subjected to mutagenesis labeled. **b** Quantification of total and cell surface levels of gB DIV mutants produced by transfected CHOs and their capacity for cell–cell fusion measured by the SRFA. All values are normalized as a percentage to WT gB. Bar charts represent *n* = 4 (*n* = 2 for SG2 staining of S589A, R592A, I594A, and ^589^AAA^594^) samples for total and cell surface gB detected using mAbs SG2 and 93k, and *n* = 28 (*n* = 8 for S589A, R592A, I594A, and ^589^AAA^594^) samples for fusion examined over two independent experiments. Error bars represent ±SEM. **c** Immunofluorescence of MeWo cells at 72 h post transfection with pOka-BACs with gB mutations. Melanoma cells were transfected with pOka-TK-GFP BACs carrying alanine substitution S589A, R592A, I594A, ^589^AAA^594^, Q596A, N597A, ^596^AA^597^, and ^592^A/^596^AA^597^. The (+) or (−) indicate whether or not virus was recovered from the transfections. Immunofluorescence staining was performed for IE62 as a marker for early infection because the TK-GFP is a late protein product during VZV replication. Scale bar (white) 100 µm. **d** Immunohistochemistry staining of plaques and their sizes for the pOka-TK-GFP gB, pOka-TK-GFP gB-TEVV5, and β23 mutants S589A, R592A, I594A, and Q596A. Scale bar (black) 1 mm. Bar charts represent *n* = 40 plaques measured over two independent experiments (Supplementary Data 2). All values were normalized to WT VZV. Error bars represent ±SEM. **e** Immunoprecipitation of the VZV gB β23 mutants from transfected CHO cells using anti gB mAbs SG2 and 93k, and western blot with anti-gB Ab 746–868. The gH control lane where CHO cells were transfected with gH-WT is shown in Fig. 6e. **f** Reducing SDS–PAGE and western blot of gB co-immunoprecipitated with gH-V5 from CHO cells transfected with the β23 mutants, gH-V5 and gL. The gB-WT control lane using the gH-WT negative control is shown in Fig. 6f. Western blots were performed using mAb to V5 (Top), anti-gB Ab 746–868 (middle), and a mAb 93k (bottom). **e**, **f** Numbers to the right of the blots are molecular weight standards (kDa). Source data are provided as a Source Data file.

To determine their biological function, gB DIV β23 residues were selected for alanine substitution, either individually or in combination and effects on gB binding to mAb 93k, gB/gH-gL-mediated membrane fusion and production of infectious VZV were evaluated. Residue S589 was used as a control because it was not a major contributor to the gB-93k interface. All of the single residue β23 substitutions except S589A significantly reduced (R592A:17%; I594A: 30%, N597A:14%) or abolished (Q596A) fusion compared to WT gB (100%) but retained the capacity to traffic to cell surfaces and bind mAb 93k (Fig. 5a, b). The quantities of cell surface gB were lower for R592A (76%), I594A (82%), and Q596A (60%) compared to WT (100%) but gB levels were not diminished to amounts that might account for the markedly reduced fusion (Fig. 5b; Supplementary Fig. 5). Although mutations at the gB-93k interface might reduce the ability of mAb 93k to bind cell surface gB, the difference in detectable levels of gB between mAb SG2 and mAb 93k could be related to prefusion and postfusion conformations of gB. Mutants that reduce fusion could prevent the binding of mAb SG2 if its epitope was only exposed in a postfusion conformation, which is in contrast to mAb 93k because its epitope is accessible in both prefusion and postfusion conformations of gB. Thus, in the context of the minimal gB/gH-gL fusion complex, the selected β23 residues might play a significant role in membrane fusion.

To explore their role in virion entry fusion, the same β23 mutations were transferred into the BAC-derived pOka-TK-GFP (designated WT) VZV (Supplementary Fig. 6A). Transfection with the WT BAC yields productive infection and spread by fusion of the virion envelope with adjacent uninfected cells, and polykaryocytes are formed due to cell fusion^36^. The gB β23 mutant BACs with single residue substitutions, R592A, I594A, and Q596A, produced infectious VZV that spread in MeWo cell monolayers, as did the S589A control, indicating a less critical role for gB-mediated virion entry and cell fusion in the presence of other VZV proteins than for fusion by the gB/gH-gL complex alone (Fig. 5b, c). Plaque sizes for Q596A but not the S589A, R592A, and I594A mutants were significantly smaller than WT VZV (Fig. 5d, Supplementary Spreadsheet 2). However, mutations that significantly reduced fusion (R592A, I594A, and Q596A) all produced significantly smaller plaques compared to S589A when incorporated into the VZV genome. This differential reflected the findings of an increase (S589) or decreases (R592A, I594A, and Q596A) in fusion, suggesting that the gB point mutations had subtle effects on VZV replication. N597A was the only single residue mutation in β23 that significantly reduced cell fusion and prevented infection of neighboring cells. This effect was unrelated to glycosylation because the N597 was not part of an NXS/T N-linked glycosylation motif. Interestingly, this residue is preserved within the gB amino acid sequence NMSR in alphaherpesviruses, suggesting a conserved function.

In contrast to the single residue substitutions, combined substitutions in β23, including ^589^AAA^594^ (triple substitution: S589A/R592A/I594A), ^596^AA^597^, and ^592^A/^596^AA^597^, prevented both gB-dependent fusion and productive infection (Fig. 5b, c). Of these, the ^589^AAA^594^ mutant had less cell surface amounts of gB (38%) and was the only mutant with significantly reduced quantities of total gB (66%) compared to WT. However, other mutations with cell surface and total gB quantities in this range have been compatible with infection, suggesting that the fusion and infection deficiency was a direct effect of ^589^AAA^594^ on gB function (Fig. 5c; Supplementary Fig. 5). Notably, transfection of the three β23 mutant BACs that had combined substitutions produced single cells that expressed the VZV immediate early protein 62 (IE62) and TK-GFP (late protein). Spread to adjacent cells and syncytia formation was prevented by these substitutions, defining their importance for the fusogenic functions of gB during VZV infection.

All of the gB β23 mutants were immunoprecipitated with either mAbs 93k or SG2, under conditions to detect conformational epitopes (Fig. 5e). The quantity of the ^589^AAA^594^ mutant was reduced for 93k compared with SG2, supporting the cryo-EM data that gB β23 contributes significantly to the gB-93k interface. The gB/gH-gL fusion complex was immunoprecipitated using mAb 93k or an anti-V5 mAb to bind gH-V5 (Supplementary Fig. 7). As mAb 93k binds to gB within DIV, these data eliminated DIV as a region necessary for the interaction of gB with gH-gL. Importantly, none of the gB β23 substitutions prevented immunoprecipitation of the gB/gH-gL complex (Fig. 5f). In contrast to immunoprecipitation, mAb 93k did not bind to the gB R592A, I594A, or ^589^AAA^594^ mutants in western blots and was reduced but not abolished for the Q596A, N597A, or ^596^AA^597^ mutants, suggesting that R592 and I594 contributed more substantially to the gB-93k interaction.

### Domain IV β30 residues are critical for fusion function

**Fig. 6:**
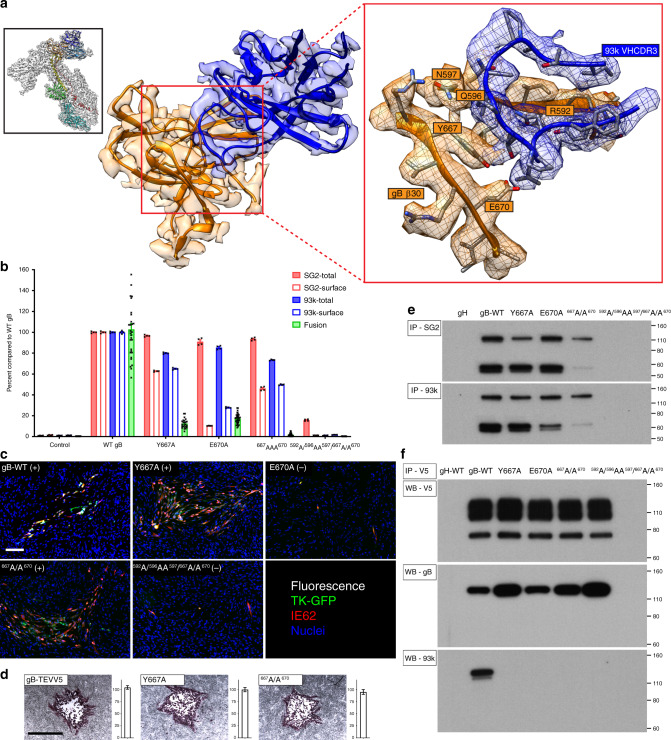
Domain IV β30 residues are critical for fusion function. **a** Near atomic structure of the mAb 93k binding site at VZV gB β30. The orientation of the complete gB-93k map is shown in the small box in the top left-hand corner colored as for Fig. 1e. A portion of the cryo-EM map for gB domain IV chain A (orange) and the bound 93k Fab (blue) are shown. The red box on the right is a magnified view of β30 with the amino acid side chains on VZV gB shown and those subject to mutagenesis labeled. **b** Quantification of total and cell surface levels of gB DIV mutants produced by transfected CHOs and their capacity for cell–cell fusion measured by the SRFA. All values are normalized as a percentage to WT gB. Bar charts represent *n* = 4 samples for total and cell surface gB detected using mAbs SG2 and 93k, and *n* = 24 (*n* = 36 for E670A) samples for fusion examined over two independent experiments. Error bars represent ±SEM. **c** Immunofluorescence of MeWo cells at 72 h post transfection with pOka-BACs with gB mutations. Melanoma cells were transfected with pOka-TK-GFP BACs carrying alanine substitutions Y667A, E670A, ^667^A/A^670^, and ^592^A/^596^AA^597^/^667^A/A^670^. The (+) or (−) indicate whether virus was recovered or not, respectively, from the transfections. Immunofluorescence staining was performed for IE62 as a marker for early infection because the TK-GFP is a late protein product during VZV replication. Scale bar (white) 100 µm. **d** Immunohistochemistry staining of plaques and their sizes for the pOka-TK-GFP gB, pOka-TK-GFP gB-STEVV5, and β30 mutants Y667A and ^667^A/A^670^. Scale bar (black) 1 mm. Bar charts represent *n* = 40 plaques measured over two independent experiments. All values were normalized to WT VZV (see Fig. 5d). Error bars represent ±SEM. **e** Immunoprecipitation of the VZV gB β23 mutants from transfected CHO cells using anti gB mAbs SG2 and 93k, and western blot with anti-gB Ab 746–868. The gH lane is a control where CHO cells were transfected with gH-WT. **f** Reducing SDS–PAGE and western blot of gB co-immunoprecipitated with gH-V5 from CHO cells transfected with the β23 mutants, gH-V5 and gL. The first gB-WT lane is a control lane using gH-WT. Western blots were performed using mAb to V5 (Top), anti-gB Ab 746–868 (middle), and a mAb 93k (bottom). **e**, **f** Numbers to the right of the blots are molecular weight standards (kDa). Source data are provided as a Source Data file.

Based on the cryo-EM structure, interface residues Y667 and E670 located within gB DIV β30 were also evaluated for their contribution to gB fusion function (Fig. 6a, b). Single substitutions, Y667A and E670A, reduced fusion (<20% of WT gB) and a double substitution, ^667^A/A^670^, abolished fusion. The limited cell surface quantity of the E670A mutant (30% of WT gB) might contribute to its reduced fusion capacity (Fig. 6b; Supplementary Fig. 8). Y667A and ^667^A/A^670^ mutant BACs both yielded infectious virus that spread to adjacent cells whereas the E670A mutant did not (Fig. 6c; Supplementary Fig. 6B). The plaque sizes for the Y667A and ^667^A/A^670^ VZV mutants were similar to both WT and S589A (Fig. 6d). However, when substitutions of the β23 and β30 interface residues were combined in the ^592^A/^596^AA^597^/^667^A/A^670^ mutant, gB expression was not affected but gB/gH-gL fusion was undetectable and VZV spread was prevented (Fig. 6b, c, f).

The single Y667A and E670A mutations and the dual, ^667^A/A^670^, substitutions reduced but did not abolish mAb 93k binding as assessed by immunoprecipitation of gB (Fig. 6e). However, mAb 93k did not bind to the Y667A, E670A, and ^667^A/A^670^ mutants in western blots, corroborating their role at the gB-93k interface. The combined β23/β30 mutant, ^592^A/^596^AA^597^/^667^A/A^670^, was not immunoprecipitated with mAb 93k, verifying the significance of these gB residues for mAb 93k binding (Fig. 6e, f). In addition, the failure of SG2 to immunoprecipitate this mutant confirmed that gB DIV residues contribute to the SG2 epitope. Similar to β23, the β30 substitutions alone did not prevent the co-immunoprecipitation of gB with gH-gL, showing that this region of gB DIV was not essential for the formation of the fusion complex.

**Fig. 7:**
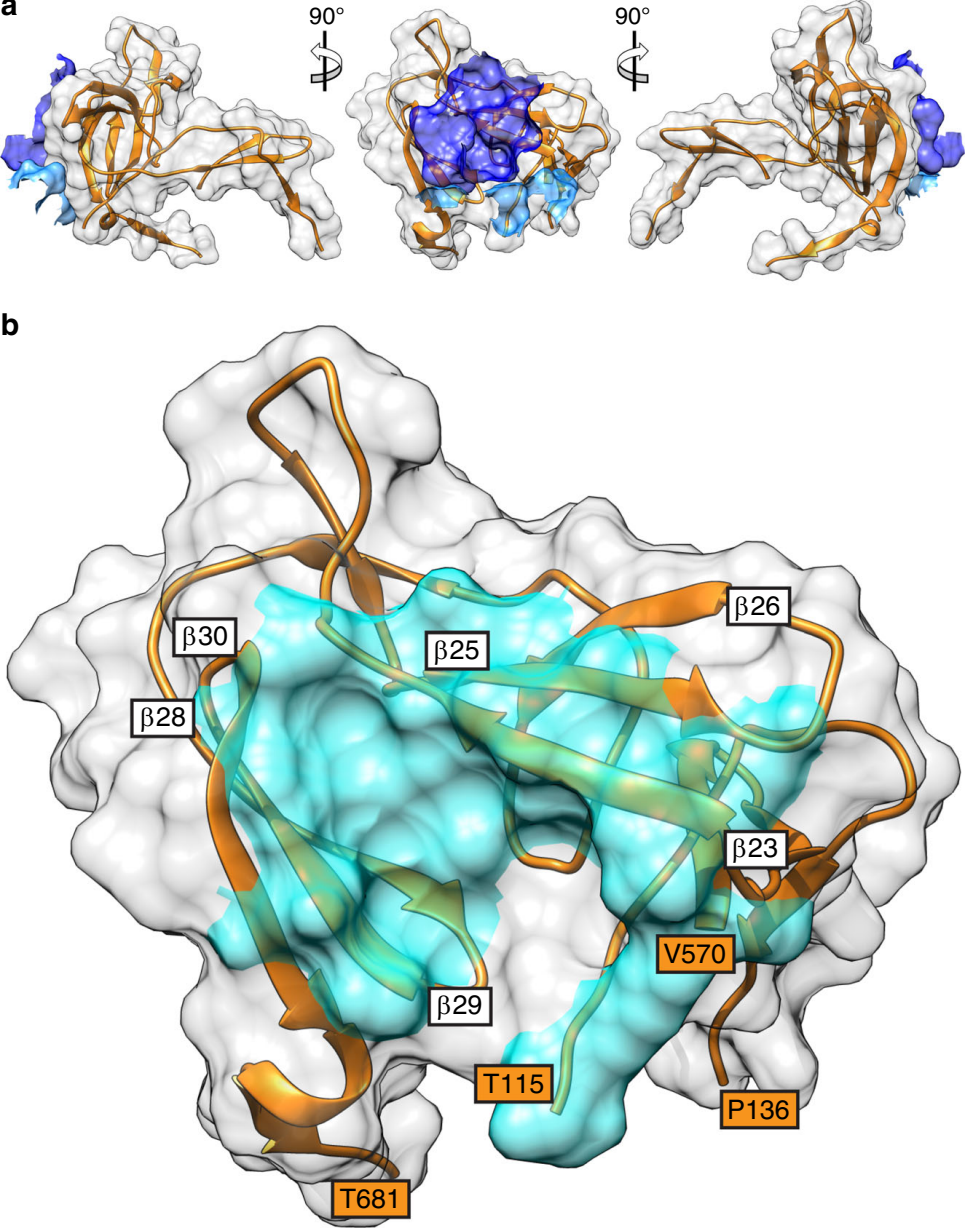
The footprint of human mAb 93k on VZV gB DIV. **a** Surface rendering (gray) of VZV gB DIV (orange ribbon diagram) and the mAb 93k residues (dark blue – VH chain; light blue – VL chain) that form molecular contacts at the gB-93k interface. **b** Surface rendering of the mAb 93k footprint (cyan) on gB DIV (gray surface; orange ribbon diagram). The amino acids that demarcate the boundaries of gB DIV are shown in orange boxes. The beta strands β23, 25–26 and β28-30 that were used to align VZV gB with herpesvirus homologs (Fig. 8) are shown in white boxes at the NH_2_ terminus of the corresponding beta strand.

**Fig. 8:**
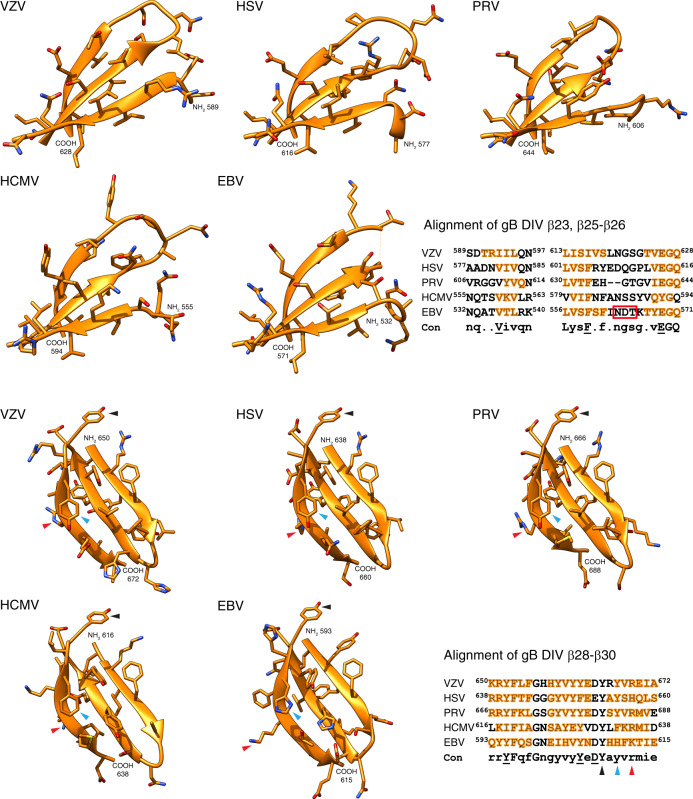
The VZV gB DIV β30 fold is conserved across the Herpesviridae. Structure alignments of VZV gB DIV encompassing β-strands 23 and 25–26, and 28–30 with those of HSV (PDB 2GUM), PRV (PDB 6ESC), HCMV (PDB 5C6T), and EBV (PDB 3FVC). The β strands are represented in ribbon format with the side chains shown. The amino acid alignments are shown in the lower right panel with each β-strand highlighted in orange. The consensus sequence (Con) shows conserved residues in upper case with those underlined showing conservations with the exception of one sequence. The red box for the EBV β23 and 25–26 alignment highlights the region not resolved in the crystal structure of 3FVC. Arrow heads highlight the conserved tyrosine (black), conserved ring structure (blue), and conserved charge (red) in β30.

The conserved conformation of VZV β30 in the structures of the gB orthologues from HSV-1, PRV, HCMV, and EBV^2,4,5,37^ implies a pan-herpesvirus role of this structural element in gB-mediated fusion, demonstrated by mAb 93k binding and at the functional level by the VZV mutagenesis data (Figs. 7 and 8). In contrast, the less conserved nature of the region corresponding to VZV β23 in the gB homologs suggests that it has evolved independently and has a virus-specific function in fusion which has diverged across the *Herpesviridae*.

## Discussion

Membrane fusion for all herpesviruses is thought to be initiated by interactions between gB and gH-gL on the virion surface to prime the gB trimer for fusion function^9^. When the fusion reaction is triggered, the fusion loops of gB DI are proposed to embed into the opposing membrane via an as yet undefined molecular process that enables gB to bridge the two membranes and bring them together in a condensation reaction. In the present study, mutagenesis based on the 2.8-Å cryo-EM structure of gB in complex with the potent neutralizing mAb 93k illuminates gB DIV as having a central role in the fusion reaction. This finding is broadly relevant as the gB fusogen is highly conserved and required for virion entry by all herpesviruses.

Since gB orthologues are classified as class III fusogens, current models of herpesvirus membrane fusion have been based on X-ray crystallography data for the prototype class III fusogen VSV G, including models of how gB orthologues transition from a putative prefusion to a postfusion conformation^6,7,9^. However, there are significant differences between these trimeric viral fusogens. Importantly, the biochemical requirements of herpesvirus gB orthologues are more complex than VSV G because interactions with the gH-gL heterodimer are necessary to prime gB for fusion^38,39^. Moreover, the quaternary structures of herpesvirus gB and VSV G DIV differ^2–6,10,11^, consistent with likely variations in the molecular mechanisms that mediate the fusion reaction. It has been challenging to determine the role of herpesvirus gB DIV in membrane fusion owing to the low-resolution cryo-EM structures for DIV in proposed prefusion conformations and its reported intrinsic flexibility^10,40–42^.

Based on the findings of the present study, we propose that the quaternary structure encompassing gB DIV, referred to as the crown^5^, remains intact during the transition of gB from prefusion conformations to its postfusion state. This is substantiated by key structural and biochemical observations. Firstly, the cryo-EM structure of the gB-93k Fab interface shows that the conformation of gB DIV was similar to that of the postfusion state^2–5^, while the fusion inhibition properties of mAb 93k authenticates the accessibility of DIV in a prefusion form of gB (Figs. 1–4). Importantly, 93k can bind to gB when it is in complex with the fusion priming heterodimer gH-gL (Supplementary Fig. 7). This implies that both gB β23 and β30, central to the 93k epitope, maintain their conformations in pre- and postfusion states of gB. As noted, the beta strand equivalent to VZV β30 remains structurally conserved in the herpesvirus gB orthologues, suggesting a conserved function in fusion (Fig. 8). Secondly, two cysteine bonds, conserved in the *Herpesviridae*^2–5^, are formed within DIV of each gB protomer at ^122^C–C^584^ and ^608^C–C^645^. The ^122^C–C^584^ bond stabilizes the N-terminal (aa115-136) and C-terminal (aa570-681) regions of gB DIV. An additional cysteine bond (^139^C–C^540^) forms a stabilizing link between the DII N-terminal linker of gB and the distal region of DIII adjacent to DIV. These cysteine bonds ensure that gB DIV forms a tertiary structure similar to the conformation of VSV G DIV, which is maintained in both the pre- and postfusion structures^6,7^. Finally, the quaternary structure of the gB crown, which is comprised of DIVs from three protomers, differs structurally from VSV G. The gB crown is elevated above DIII and each DIV from a protomer in the gB trimer co-assembles to form the crown. This contrasts with VSV G where the DIV protomers remain as individual tertiary structures adjacent to DIII to form the crown^6,7^. Within each protomer, VSV G DIV also remains in close proximity to DII in the pre- and postfusion structures whereas the herpesvirus gB DIV is located away from and opposite to DII within the gB protomer, further supporting the notion that the crown of gB orthologues remains as a single quaternary structure during the prefusion to postfusion transition.

Functional data derived from the virus-free fusion assay and VZV BAC transfection demonstrated that most DIV point mutations were tolerated in the context of VZV infection. This is consistent with previous work investigating the effects of gB point mutations on herpesvirus fusion function^37^. The exceptions in the present study were N597 and E670, which, considering the gB structure, might have roles in currently unexplored protein–protein interactions. In contrast, combined substitutions of two or more of the β23 and β30 residues reduced or abolished fusion and limited the capacity of VZV to infect cells, indicating that these residues act together to ensure that the gB structure supports its fusion function. The greater tolerance for DIV mutations in the context of VZV infection suggests that other viral proteins might act as accessory molecules to facilitate gB-dependent fusion as reported for other herpesviruses, such as HSV-1 gD, HCMV gO or UL128-131, and EBV gp42^43–45^. VZV gB might also interact with cell surface proteins as has been demonstrated for HSV-1 (PILRα), HCMV (PDGFRα), and EBV (NRP1; neuropilin 1)^46–49^, although gB domains important for these interactions have not been identified by mutagenesis or other methods. While VZV gB binding to myelin associated glycoprotein (MAG) has been described^30^, the interaction was not specific as VZV gE also bound to MAG^50^. Notably, co-immunoprecipitation of the β23 and β30 mutants demonstrated that this region of DIV was not required for gB binding to gH-gL, providing further evidence that this subdomain is present on the prefusion form of gB and the interaction with gH-gL does not mask DIV during fusion. This finding is consistent with a low-resolution structure indicating that gH-gL binds to a putative prefusion form of gB on the surface of herpesvirus particles^13^.

Neutralizing antibodies also target the HSV-1 and HCMV gB DIV, indicating that the accessible residues of prefusion forms of gB are of broad importance for herpesvirus fusion function^4,14–19^. HSV-1 was neutralized by murine mAbs, SS10 and SS63, produced in mice vaccinated with gB^15^. Although the location of the SS63 epitope could not be defined further than DIV, truncation mutants of gB and low-resolution mapping by negative stain EM approximated SS10 binding to HSV-1 gB residues 640–670^14,15^. While the SS10 epitope was not reported to overlap with VZV gB β23, HSV-1 gB residues 640–670 are located in the region corresponding to VZV gB β30, which is conserved in herpesvirus gB orthologues (Figs. 7 and 8). Neutralizing Ab epitopes have been mapped to five antigenic domains (AD) of HCMV gB^4,16–18^. Of these, DIV contains AD-1 and AD-2 (within the first 80 N-terminal residues), DII contains AD-4, DI contains AD-5, and AD-3 is at the C-terminus of full-length gB. For HCMV, AD-1 is an immunodominant region for neutralizing antibodies^51^. Thus, these studies further support the role of DIV in gB-mediated membrane fusion initiation for both alpha- and beta-herpesviruses.

In summary, this study has demonstrated the power of a cryo-EM-based structure-function approach that solves the structure of a fusogenic viral glycoprotein recovered from infected cells in complex with a neutralizing antibody, thereby providing essential information to guide the biological discovery of glycoprotein residues critical for fusion function. These data provide compelling evidence that a domain independent of the gB fusion loops is required for herpesvirus fusion and has the potential to be utilized as a novel target for antiviral therapies.

## Methods

### Reagents and resources

All reagents, consumables, and resources, including supplier information where applicable, are provided in Supplementary Table 5.

### Cells lines

All cell lines were propagated at 37 °C in a humidified atmosphere with 5% CO_2_. MeWo cells (HTB-65; ATCC) were propagated in minimal essential medium (Corning Cellgro) supplemented with 10% fetal bovine serum (FBS; Invitrogen), nonessential amino acids (100 μM; Corning Cellgro), antibiotics (penicillin, 100 U/ml; streptomycin, 100 μg/ml; Invitrogen), and the antifungal agent amphotericin B (Invitrogen). CHO-DSP1 cells previously derived from the Chinese hamster ovary (CHO) K1 cell line (CCL-61; ATCC) and express the dual split protein (DSP1) R8(1–8)^32^ were propagated using F-12K nutrient mixture with Kaighn’s modification (Invitrogen) supplemented with 10% FBS and antibiotics (penicillin, 100 U/ml; streptomycin, 100 μg/ml; Invitrogen) and maintained under puromycin selection (8 μg/ml; Invitrogen). Mel-DSP2 cells previously derived from the MeWo cell line and express the DSP2 R8(9–11)^32^ were propagated as for MeWo cells but under puromycin selection (5 μg/ml).

### Viruses

The VZV parental Oka strain was originally cloned into a bacterial artificial chromosome (BAC) and designated pPOKA-BAC-DX^52^. The recombinant virus pOka-TK-GFP (pOka-rTK) was generated in a previous study^36^. All recombinant pOka-TK-GFP VZV mutants were derived from the self-excisable BAC, pPOKA-TK-GFP-BAC-DX ΔORF31^36^. The gB-KAN cassette^53^ was digested with BstZ171 and NaeI. The 4056-bp fragment was gel-purified and used to transform electrocompetent GS1783 *Escherichia coli* carrying the pPOKA-TK-GFP ΔORF31 BAC. After red recombination, the pPOKA-TK-GFP BAC was purified using a large-construct purification kit (Qiagen). BACs were digested with Hind III to verify that spurious recombination had not occurred, and successful incorporation of ORF31 mutations were verified by sequencing the BAC directly. To generate BAC-derived VZV, 10^6^ MeWo cells seeded in six-well plates (Nunc) 24 h previously were transfected with 4 µg of the pPOKA BACs using Lipofectamine 2000 (Invitrogen) following the manufacturer’s instructions. Recombinant VZV was typically recovered at 5–10 days post-transfection. All virus stocks, pOka and gB DIV mutants, were sequenced to verify that the expected ORF31[gB] sequence was present. Briefly, DNA was extracted from infected cells using proteinase K and phenol/chloroform (Invitrogen). VZV ORF31 was amplified by PCR with KOD Extreme™ (EMD Millipore) following the manufacturer’s instructions using the oligonucleotides [31]F56625-56645/[31]R59697-59717. The PCR products were gel purified and sequenced by Sanger sequencing.

### Construction of VZV pOka-TK-GFP-gB-TEVV5

For the purification of gB from VZV-infected cells, a green fluorescent protein (GFP) expressing virus was generated that produced gB with a tag containing the tobacco etch virus protease cleavage site and a V5 epitope (TEVV5). A gB-Kan-TEVV5 shuttle vector was generated in a three-step cloning procedure. First, a gB-Kan-V5 vector was generated by amplifying two fragments from the gB-Kan vector^53^ using AccuPrime™ *Pfx* (Invitrogen) with oligonucleotides gB-AgeI/gB931 and gB-V5/M13R, purified using a QIAquick gel purification kit (QIAGEN) following the manufacturer’s instructions, and ligated into the AgeI and SpeI site of the gB-Kan vector. Secondly, two fragments were amplified from gB-Kan-V5 using AccuPrime™ *Pfx* with oligonucleotides gB-AgeI/gB_cterm_Sprotein and gB_link_TEV_link/M13R, gel purified and ligated into the AgeI/SpeI site of gB-Kan. The final step deleted the S-tag from the gB-Kan-STEVV5 to generate the gB-Kan-TEVV5 shuttle vector. Two fragments were amplified from gB-Kan-STEVV5 using AccuPrime™ *Pfx* with oligonucleotides gB-AgeI/ΔS-tag-sense and ΔS-tag-antisense/M13R, gel purified, and ligated into the AgeI and SpeI site of the gB-Kan vector. The gB-Kan-TEVV5 shuttle vector was used to reconstitute ORF31-TEVV5 into the pOka-TK-GFP-ΔORF31 BAC to generate pPOKA-TK-GFP-gB-TEVV5 and recovery of pOka-TK-GFP-gB-TEVV5 virus was performed as described in the ‘Viruses’ section.

### Construction of the VZV pOka-TK-GFP-gB-TEVV5 gB DIV mutants

Site directed mutagenesis was performed using the pCAGGs-VZV gB (pCAGGs-gB) vector for template to generate DNA fragments using AccuPrime™ *Pfx*. For the single alanine substitutions at S589A, R592A, and I594A, DNA fragments were amplified using the oligonucleotide combinations pCAGGs-gB-XmaI-sense/S589A-antisense (S589A), S589A-sense/pCAGGs-gB-AgeI-antisense, pCAGGs-gB-XmaI-sense/R592A-antisense (R592A), pCAGGs-gB-XmaI-sense/I594A-antisense (I594A), and pCAGGs-gB-3617-sense/pCAGGs-gB-AgeI-antisense. For the combined alanine substitutions at S589A/R592A/I594A (^589^AAA^594^), DNA fragments were amplified using the oligonucleotides pCAGGs-gB-XmaI-sense/589AAA594-antisense and 589AAA594-sense/pCAGGs-gB-AgeI-antisense. For alanine substitutions at Q596, N597, and ^596^QN^597^, DNA fragments were amplified using the oligonucleotide combinations pCAGGs-gB-XmaI-sense/Q596A-antisense (Q596A), pCAGGs-gB-3623-sense/pCAGGs-gB-AgeI-antisense, pCAGGs-gB-XmaI-sense/pCAGGs-gB-3622-antisense, N597A-sense/pCAGGs-gB-AgeI-antisense (N597A), pCAGGs-gB-XmaI-sense/Q596A-antisense, and N597A-sense/pCAGGs-gB-AgeI-antisense (^596^QN^597^). All DNA fragments were gel purified and digested with the appropriate restriction enzymes for ligation into the XmaI/AgeI site of pCAGGs-gB. To generate the ^592^A/^596^QN^597^ substitutions the pCAGGs-gB-R592A was used as template to amplify DNA fragments using oligonucleotides pCAGGs-gB-XmaI-sense/R592A-Q596A-antisense and N597A-sense/pCAGGs-gB-AgeI-antisense. The two DNA fragments were gel purified and digested with the appropriate restriction enzymes for ligation into the XmaI/AgeI site of pCAGGs-gB. For the alanine substitution Y667A, E670A and ^667^A/A^670^, DNA fragments were amplified using the oligonucleotide combinations pCAGGs-gB-MluI-sense/Y667A-antisense (Y667A), E670A-sense/pCAGGs-gB-AgeI-antisense (E670A), pCAGGs-gB-MluI-sense/Y667-antisense and V668-sense/pCAGGs-gB-AgeI-antisense. All DNA fragments were gel purified and digested with the appropriate restriction enzymes for ligation into the MluI/AgeI site of pCAGGs-gB. To generate the ^592^A/^596^QN^597^/^667^A/A^670^ substitutions the pCAGGs-gB-592A/596AA597 was used as template to amplify DNA fragments using oligonucleotides pCAGGs-gB-MluI-sense/Y667A-antisense (Y667A) and E670A-sense/pCAGGs-gB-AgeI-antisense (E670A). All the pCAGGs vectors were sequenced to verify that only the specific mutations were incorporated and spurious mutations from the PCR had not been incorporated.

The ^589^AAA^594^, ^596^AA^597^, ^592^A/^596^AA^597^, and ^592^A/^596^AA^597^/^667^A/A^670^ combined and single mutations were transferred into the gB-Kan-TEVV5 shuttle vector by restriction digest of the pCAGGS-gB mutant vectors and cloned into the NdeI/AgeI site of the gB-Kan-TEVV5. All the gB-Kan-TEVV5 vectors were sequenced to verify that only the specific mutations were incorporated and spurious mutations from the PCR had not been incorporated. The gB-Kan-TEVV5 shuttle vectors were used to reconstitute ORF31-TEVV5 DIV mutants into the pOka-TK-GFP-ΔORF31 BAC to generate pPOKA-TK-GFP-gB-TEVV5 DIV mutant BACs and recovery of pOka-TK-GFP-gB-TEVV5 DIV mutant viruses was performed as described in the ‘Viruses’ section. Critically, sequencing of virus stocks confirmed that none of the gB β23 mutants had unexpected nucleic acid substitutions in ORF31 (Supplementary Fig. 6).

### Purification of native full-length gB from VZV-infected cells

MeWo cells were infected with the pOka-gB-TEVV5 virus and replication was allowed to proceed until cytopathic effect was observed across 90% of the cell monolayer. Infected cells were scrapped into ice cold PBS and pelleted at 424 RCF for 5 mins. Cells were lysed in glycoprotein extraction buffer (0.1 M Tris-base[pH7.2], 0.1 M NaCl, 5 mM KCL, 1 mM CaCl_2_, 0.5 mM MgCl_2_, 1% sodium deoxycholate, and 1% NP40) plus an EDTA-free protease inhibitor cocktail (Roche, CA, USA)^54^. Cell lysates were clarified at 3000 RCF for 10 mins and 5 ml of clarified lysate was incubated with 250 μl of anti-V5 agarose beads (Sigma) for 2 h at room temperature. The beads were washed extensively in PBS + 0.1% Triton and a final wash with PBS then incubated with PBS containing TEV protease (55 µg/ml) for 20 h +4 °C. The TEV cleaved gB was eluted from the beads with TBS pH7.4 containing 1 mg/ml lauroylsarcosine (Sigma) and 1 mg/ml Amphipol 8–35 (Anatrace). Buffer exchange into TBS pH7.4 and 1 mg/ml Amphipol 8–35 using Amicon® Ultra-4 centrifugation filters with a 100 kDa cutoff (Millipore). The concentration of Amphipol 8–35 was brought up to 35 mg/ml and incubated at room temperature for 4 h then Bio-Beads™ SM-2 (Bio-Rad) were added and incubated for 16 h at 4 °C. The purified native, full-length gB was resolved on either Native PAGE or denaturing SDS–PAGE and either stained with Coomassie (Native PAGE) or Gel Code Blue (SDS–PAGE) following the manufacturer’s instructions. To determine that the purified protein was native, full-length gB western blot was performed by transferring proteins to Immobilon-P membranes (Millipore Biosciences, Temecula, CA) and blocked with 5% BSA. The mouse mAb SG2, the human mAb 93k and a rabbit polyclonal antiserum, 746–868, which recognizes the peptide sequence ^833^PEGMDPFAEKPNAT^846^ in the cytoplasmic region of pOka gB^33^, were used to detect gB. Horse radish peroxidase conjugated antibodies that detect either mouse, human, or rabbit IgG (GE Healthcare Bio-Sciences Corp., Piscataway, NJ) were used and HRP activity detected using ECL plus (GE Healthcare Bio-Sciences Corp., Piscataway, NJ). The native, full-length gB was further purified on a Superose-6 column (GE Healthcare Life Sciences) into TBS pH7.4 to remove aggregates.

### Isolation of mAb 93k and preparation of 93k Fab

B-lymphocytes from a VZV immune individual were used to generate triomas that secrete antibodies that cross reacted with VZV gB and had neutralizing activity against 11 clinical isolates^55^. Triomas that secreted gB reactive antibodies were subcloned by limiting dilution resulting in clone 93kA9. The 93k Fab coding sequences for the variable heavy (VH) chain and variable light (VL) were subsequently sequenced and cloned into the pRS5a mammalian expression vector (Novartis AG, Basel, Switzerland), which expresses a generic constant region of IgG1 heavy chain (HC) and light chain (LC). The complete heavy chain has a cleavable double strep tag at the C-terminus. Fab fragments were generated from mAb 93k then purified by affinity and size-exclusion column.

### Preparation of mAb 93k Fab fragments bound to the native full-length VZV gB

Native full-length VZV gB was purified from infected cells as described in the ‘Purification of native full-length gB from VZV-infected cells’ section except the mAb 93k Fab fragments were added in molar excess immediately after the TBS pH7.4 and 1 mg/ml Amphipol 8–35 buffer exchange. The native full-length gB plus Fab fragments were incubated overnight at +4 °C on a rotary mixer. The complexes were concentrated using Amicon Ultra 10 kDa filter units following the manufacturer’s instructions. The concentration of Amphipol 8–35 was brought up to 35 mg/ml and incubated at room temperature for 4 h then Bio-Beads™ SM-2 (Bio-Rad) were added and incubated for 16 h at 4 °C. The purified native, full-length gB-Fab complexes were evaluated by Native PAGE and purified on a Superose-6 column (GE Healthcare Life Sciences) into TBS pH7.4 to remove aggregates.

### Grid freezing

Lacey carbon copper 400 mesh grids with an ultrathin layer of carbon or Quantifoil R 1.2/1.3 gold 300 mesh grids were used for specimen freezing. EM grids were glow discharged for 25 s. To each grid, 3 µl of purified protein was dispensed and immediately plunge frozen into liquid ethane using a Leica EM GP. Optimum chamber humidity and blotting times were determined empirically for each sample and ranged from 95 to 99% and 1.8–2.5 s.

### Cryo-EM data collection

Micrographs for native, full-length VZV gB in complex with 93k Fab fragments were captured on a 300-kV Titan Krios (FEI) controlled by SerialEM^56^ to automate the data collection procedure. Movie data (11,283 total stacks) were captured with a Gatan K2 Summit (5 μm/pixel) in counted mode with a dose rate of ~1.335 e^−^/Å^2^/s per frame and 200 millisecond exposure time per frame and 12-s total exposure time at a nominal magnification of ×130,000 and a pixel size of 1.06 Å/pixel on the specimen. The defocus range was 1.5–2.0 µm.

### Map reconstruction of full-length VZV gB in complex with mAb 93k Fab

The motion correction and damage compensation for all movie-mode data were performed using MotionCor2^57^. CTFFIND4 was used to estimate the contrast transfer function parameters^58^. Initially, the first 100 micrographs were selected to box out particle images using EMAN2’s e2boxer.py^59^, followed by Relion’s^60,61^ 2D classification which generated a set of 2D class averages. The good 2D class averages were selected as templates to box out the particle images from all micrographs using Relion’s auto-picking. EMAN2’s e2initialmodel.py^59^ or Relion’s 3D initial model was utilized to build the initial model. A couple of Relion’s 2D classifications were first performed to remove junk, and the good classes were selected from 3D classification to do the final 3D auto-refine, for which 856,068 particles were selected. The C3 symmetry was imposed during the 3D auto-refine of Relion.

### Structure and visualization of the VZV gB-93k complex

The resolution of the cryo-EM map and model of gB-93k was determined using Fourier shell correlation overall resolution estimate^62^. A protomer model was built by fitting our X-ray structure (PDB 6VLK; unpublished) and the 93k VH and VL chains were built de novo. The gB-93k structure models were generated and refined using Coot^63^ and Phenix^64–67^. ResMap was used to calculate local resolution variation^34^. A newly developed Q scoring tool was applied to calculate feature resolvability^35^. Interactions between amino acids were calculated using the Find Contacts tool in UCSF Chimera 1.13.1 using the default settings^68^. Surface electrostatic potential was calculated using APBS (Adaptive Poisson-Boltzmann Solver)^69^. All images and movies were generated using the Animation tool in UCSF Chimera 1.13.1.

### Cell-free VZV neutralization assay

Cell-free VZV stocks were prepared as described^70^. MeWo cells in 100 mm culture dishes infected with pOka-TK-GFP were washed with cold PBS then incubated at room temperature with PBS + 0.1% EDTA. The cells were dislodged by pipetting, centrifuged at 424 RCF for 5 mins then resuspended in PSGC buffer (PBS + 145 M sucrose + 6 mM L( + )-glutamic acid + 10% FBS). The resuspended cells were transferred to a Dounce homogenizer (KONTES^®^) and disrupted with 15 strokes of pestle A and 15 strokes of pestle B. The resulting homogenate was centrifuged at 3000 RCF to removed cell debris and the supernatant was stored in 1 ml aliquots under liquid nitrogen. The titers of cell-free VZV were typically in the 3.5 log_10_/ml range. To test antibodies for VZV neutralization capabilities, 100 pfu of cell-free pOka-TK-GFP was incubated with 10 μg antibody (93k, SG2 or 206) at room temperature for 30 mins. MeWo cells seeded 24 h previously at 1 × 10^5^/cm^2^ in 12-well plates were inoculated with the pOka-TK-GFP/antibody mixtures and incubated for 24 h. The media was changed, and the plates were incubated for a further 72 h then fixed with 4% paraformaldehyde. Plaques were detected by immunohistochemical staining using an anti-VZV mixed mouse mAb followed by detection with a streptavidin conjugated goat anti-mouse IgG (Jackson ImmunoResearch Laboratories, Inc., West Grove, PA) and alkaline phosphatase conjugated avidin (Jackson ImmunoResearch Laboratories, Inc., West Grove, PA). Enzyme activity was detected using a fast red substrate (0.1MTris - pH 8.0, 5 µM Naphthol AS-Mx phosphate (Sigma), 80 µM Fast Red TR (Sigma)).

### Quantitation of cell surface gB for the DIV mutants

CHO-DSP1 cells (8 × 10^5^ cells/well) in six-well plates were transfected with 5 μg WT or mutant pCAGGS-gB expression vectors. Cells were dislodged at 24 h post transfection using an enzyme-free cell dissociation buffer (Life Technologies, Grand Island, NY), washed with PBS then fixed with 1% paraformaldehyde. The fixed cells were washed with PBS then resuspended in FACS staining buffer (DPBS (Dulbecco’s Phosphate-Buffered Saline; Cellgro, Manassas, VA) with 0.2% IgG-free BSA (Jackson ImmunoResearch, West Grove, PA) and 0.1% NaN3 (Sigma Aldrich, St. Louis, MO)) for cell surface staining with anti-VZV gB mAb SG2-2E6 or 93k. A donkey anti-mouse IgG-Alexa Fluor 555 antibody (SG2-2E6) or goat anti-human IgG-Alexa Fluor 488 antibody (93k) (Life Technologies, Grand Island, NY) was used to detect bound anti-VZV gB mAb. Total gB expression was determined by using the same staining protocol except cells were permeabilized using Cytofix/Cytoperm (BD Biosciences, San Jose, CA) before adding the primary antibody and during the staining procedure. Stained cells were analyzed using a FACSCalibur with CellQuest Pro (BD Biosciences, San Jose, CA). FlowJo (TreeStar, Ashland, OR) was used to determine the quantity of total and cell surface gB on the transfected cells. The quantities for gB mutants were normalized to WT gB, which was set at 100%. Experiments were performed with at least two gB mutant clones, each tested in duplicate.

### VZV stable reporter fusion assay

The stable reporter fusion assay for the VZV glycoproteins gB/gH-gL has been reported previously^32^ but was adapted for use with a 96-well plate format. CHO-DSP1 cells seeded at 8 × 10^5^ per well in six-well plates 20 h previously were transfected with 1.6 μg each of pCAGGs-gB, pME18s-gH[TL], and pCDNA-gL plasmids with Lipofectamine 2000 following the manufacturer’s instructions. At 6 h post transfection, the transfected CHO-DSP1 cells were trypsinized, collected by centrifugation at 424 RCF, and resuspended in 1 ml of medium, of which 250 μl of cells were mixed with 0.75 ml of Mel-DSP2 cells at 10^6^ cells/ml. To test fusion inhibition properties of mAbs, 93k, SG2 and 206, 10 µg of antibody was added to the cell mixtures. The cells were mixed by inversion and 75 µl of the suspension was dispensed to at least triplicate wells of 96-well blacked sided optical bottom plates culture plates (Thermo Scientific). At 40 h post seeding, 50 µl membrane permeable coelenterazine-H (5 μM, Nanolight Technology) substrate for five mins at room temperature. Fusion was quantified by measuring luminescence using a Synergy H1 Multi-mode reader (Biotek). A minimum of two clones were tested in duplicate experiments.

### Immunofluorescence staining of MeWo cells

To each well of a 12-well plate (Nunclon™ Delta Surface; Thermo Scientific) a sterile 18 mm coverslip (Fisher Scientific) was placed and 2 ml of MeWo cells at 2 × 10^5^/ml was dispensed and incubated overnight. MeWo cells were transfected with 2ug of pPOKA-TK-GFP BACs carrying gB mutants using Lipofectamine 2000 following the manufacturer’s instructions. At 72 h post transfection, the media was aspirated, the coverslips washed with PBS and fixed with 4% paraformaldehyde for 10 mins. Immunofluorescence was performed by blocking the cells with PBS + 10% normal donkey serum (NDS) + 0.1% Triton X-100 then adding a mouse mAb to the immediate early protein IE62 in PBS + 1% NDS + 0.1% Triton X-100. The anti-IE62 mAb was detected with the donkey anti-mouse IgG-Alexa Fluor 555 and nuclei were stained with Hoechst 33342 in PBS + 1% NDS + 0.1% Triton X-100. Coverslips were mounted on glass slides (Selectfrost; Fisher Scientific) using Fluoromount-G (SouthernBiotech) and a minimum of five images were captured for each transfection using a Keyence fluorescence microscope using a ×20 objective.

### Quantitation of plaque sizes for the VZV gB DIV mutants

MeWo cells were seeded at 10^6^ cells/well 24 h prior to inoculation with 50 pfu of either wild type pOka or gB DIV mutants. Each well of the six-well plate was fixed at 4 days post inoculation with 4% formaldehyde and stained by immunohistochemistry. Images of stained plaques (*n* = 40) were digitally captured, the stained plaque was outlined, and the area (mm^2^) was calculated using ImageJ (National Institute of Mental Health). Statistical analyses were performed using Prism (GraphPad Software).

### Immunoprecipitation of VZV gB DIV mutants

CHO-DSP1 cells seeded in six-well plates were transfected with 5 µg/well of pCAGGS-gB vectors carrying the DIV mutations using Lipofectamine 2000 following the manufacturer’s instructions. At 24 h post transfection cells were lysed with glycoprotein lysis buffer, the same buffer used for the purification of native, full-length VZV gB, and snap frozen in liquid nitrogen and stored at −20 °C. The SG2 or 93k mAbs were cross-linked to immobilized protein A (Pierce, Rockford, IL)^71^. Each 20 µg of mAb was incubated with 30 µl protein A beads for 1 h at room temperature on a rotary mixer. The beads were washed with DPBS then mAbs were cross-linked to the beads with 0.2 M sodium borate [pH9.0] and 20 mM DMP for 30 mins. The cross-linking reaction was quenched with 0.2 M NaCl and 0.2 M ethanolamine [pH 8.0] for 2 h at room temperature. The cross-linked beads were washed with DPBS. Lysates from the pCAGGs-gB transfected CHO-DSP1 cells were divided equally and incubated overnight at +4 °C with either the SG2 or 93k cross-linked beads. The beads were washed extensively with DPBS + 0.1% Triton X-100 and a final wash of DPBS to remove the Triton X-100. Bound proteins were eluted into sodium dodecyl sulfate (SDS) sample buffer (Bio-Rad) containing 5% 2-mercaptoethanl (Sigma) by incubating the beads at 100 °C for 5 min. Denatured samples were resolved on SDS-polyacrylamide gel electrophoresis precast gels (Bio-Rad, Hercules, CA) and western blot was performed using the 746–868 rabbit poly clonal IgG.

### Immunoprecipitation of VZV gB DIV mutants in complex with gH-gL

CHO-DSP1 cells were transfected as described in the previous section with pCAGGS-gB vectors carrying the DIV mutations, pME18s-gH[V5] and pCDNA3.1-gL (1.6 µg of each vector). At 24 h post transfection cells were lysed with glycoprotein lysis buffer and snap frozen in liquid nitrogen and stored at −20 °C. The gB/gH-gL complexes were immunoprecipitated with anti-V5 agarose (Sigma). Wash steps, protein elution, and SDS–PAGE were performed as outlined in the previous section. Western blots were performed using either mouse anti-V5 tag (Bio-Rad), 746–868 rabbit poly clonal IgG, or mAb 93k.

### Statistics and reproducibility

All quantitative data were analyzed with two-way ANOVA to determine statistical significance using Prism (GraphPad Software). All statistical analyses are presented in the Source Data file. Images of gB-93k class averages in Fig. 2a are representative of 23 classifications. Confocal micrographs in Figs. 5c and 6c are representative images of *n* = 10 from two independent experiments. Images of gels and western blots in Figs. 1a, 5e, f, and 6e, f and Supplementary Figs. 2a, b and 7 are representative of at least two independent experiments.

### Reporting summary

Further information on research design is available in the Nature Research Reporting Summary linked to this article.

## Supplementary information

Supplementary Information

Peer Review File

Reporting Summary

Description of Additional Supplementary Files

Supplementary Data 1

Supplementary Data 2

Supplementary Movie 1

Supplementary Movie 2

Supplementary Movie 3

Supplementary Movie 4

## Data Availability

Maps and models have been deposited in the Electron Microscopy Data Bank with accession code EMD-21247 and the Protein Data Bank with accession code 6VN1. Source data are provided with this paper. All primary data will be provided by the corresponding author upon reasonable request. Source data are provided with this paper.

## Source data

Source Data
