# Publisher Correction: A glycoprotein B-neutralizing antibody structure at 2.8 Å uncovers a critical domain for herpesvirus fusion initiation

**DOI:** 10.1038/s41467-020-18385-w

**Published:** 2020-08-28

**Authors:** Stefan L. Oliver, Yi Xing, Dong-Hua Chen, Soung Hun Roh, Grigore D. Pintilie, David A. Bushnell, Marvin H. Sommer, Edward Yang, Andrea Carfi, Wah Chiu, Ann M. Arvin

**Affiliations:** 1 Department of Pediatrics, Stanford University School of Medicine, Stanford, CA 94305 USA; 2 GSK Vaccines, Cambridge, MA 02139 USA; 3 Structural Biology, Stanford University School of Medicine, Stanford, CA 94305 USA; 4 Department of Biological Sciences, Institute of Molecular Biology & Genetics, Seoul National University, Seoul, 08826 Korea; 5 Bioengineering, Stanford University School of Medicine, Stanford, CA 94305 USA; 6 Microbiology and Immunology, Stanford University School of Medicine, Stanford, CA 94305 USA; 7 Division of Cryo-EM and Bioimaging SSRL, SLAC National Accelerator Laboratory, Menlo Park, CA 94025 USA; 8 Present Address: Syros Pharmaceuticals, Inc., Cambridge, MA 02140 USA; 9 Present Address: Moderna, Cambridge, MA 02139 USA

**Keywords:** Antibodies, Herpes virus, Viral membrane fusion, Virus-host interactions, Cryoelectron microscopy

Correction to: *Nature Communications* 10.1038/s41467-020-17911-0, published online 18 August 2020.

The original version of this Article contained errors in the author affiliations.

Yi Xing and Andrea Carfi were incorrectly associated with ‘Division of Cryo-EM and Bioimaging SSRL, SLAC National Accelerator Laboratory, Menlo Park, CA 94025, USA’ instead of the correct ‘Syros Pharmaceuticals, Inc., Cambridge, MA 02140, USA’ and ‘Moderna, Cambridge, MA 02139, USA’, respectively.

This has now been corrected in both the PDF and HTML versions of the Article.

