# Supplementary material for: A glycoprotein B-neutralizing antibody structure at 2.8 Å uncovers a critical domain for herpesvirus fusion initiation
Source: Nat Commun. 2020 Aug 18;11:4141. doi: 10.1038/s41467-020-17911-0 (PMC7435202; doi:10.1038/s41467-020-17911-0)
Supplement: Supplementary file 1 — Supplementary Information [file 41467_2020_17911_MOESM1_ESM.pdf]

Supplementary Information

For

A Glycoprotein B-Neutralizing Antibody Structure at 2.8Å

Uncovers a Critical Domain for Herpesvirus Fusion Initiation.

Oliver et al.

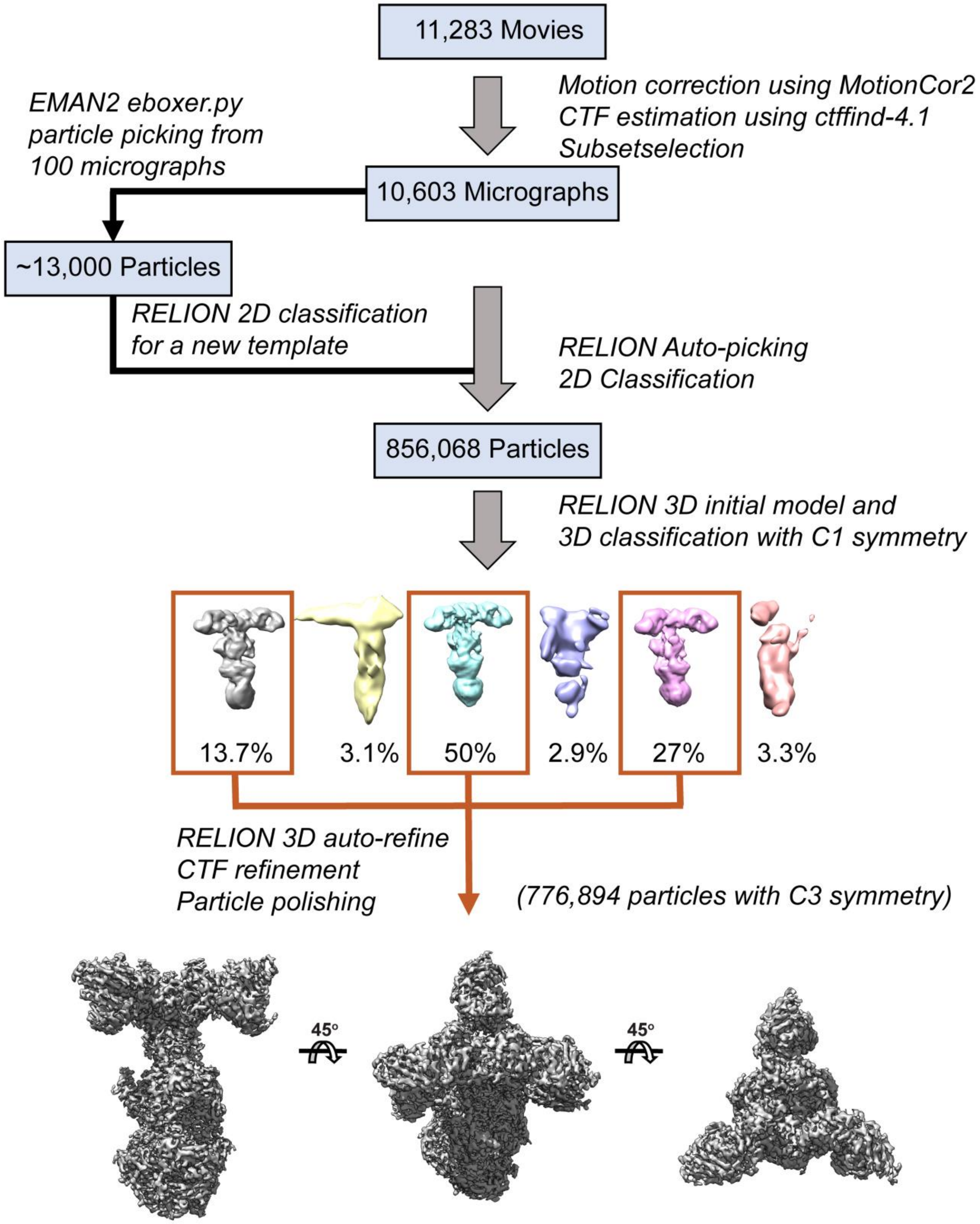

Supplementary Figure 1. Classification scheme of particles identified in cryo-EM micrographs of purified native VZV gB in complex with 93k Fab fragments.

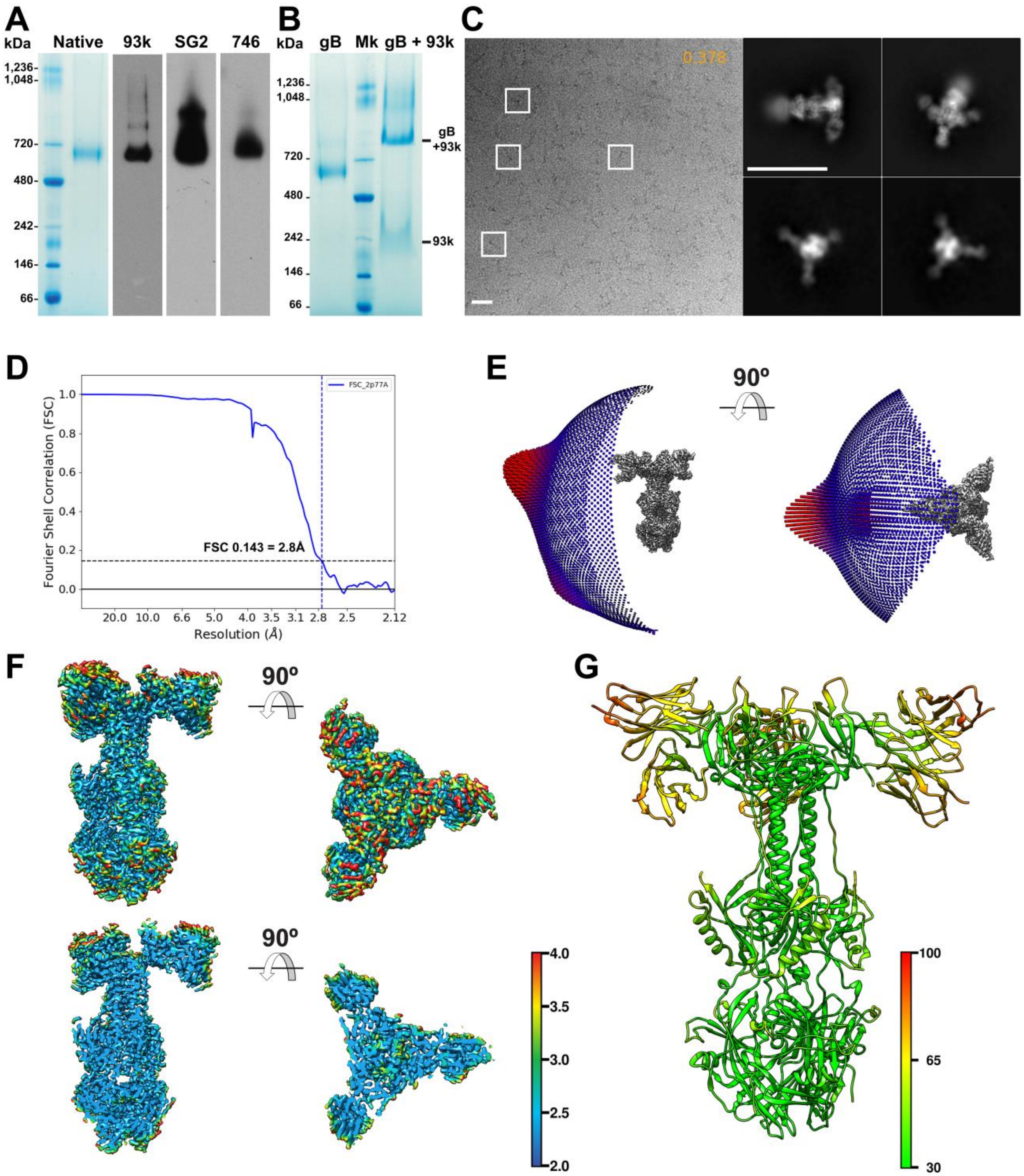

Supplementary Figure 2. Near atomic resolution (2.8Å) structure of 93k Fab fragments bound to gB purified from VZV infected MeWo cells. A – Native PAGE of purified native VZV gB and western blots with VZV gB specific antibodies 93k (Hu mAb), SG2 (Mu mAb) and 746-868 (Rb IgG). Molecular weights of the protein standards are given to the left of the gel (kDa). B – Native PAGE of purified VZV gB and the formation of the gB-93k Fab complex. C – Single particle cryo-EM micrograph of purified VZV gB in complex with 93k Fab fragments post size exclusion chromatography in vitreous ice on a lacey carbon grid with four

representative 2D class averages. Scale bars (white) 20nm. D and E – Fourier shell correlation plot (E; FSC 0.143 = 2.8Å) and Euler angle distribution (E) of VZV gB-93k particles included in the 3D reconstruction. F – Local resolution of the cryo-EM map quantified using ResMap [1]. G – A model of the VZV gB-93k complex represented in ribbons and colored according to B-factors derived from structure refinement using Phenix [2].

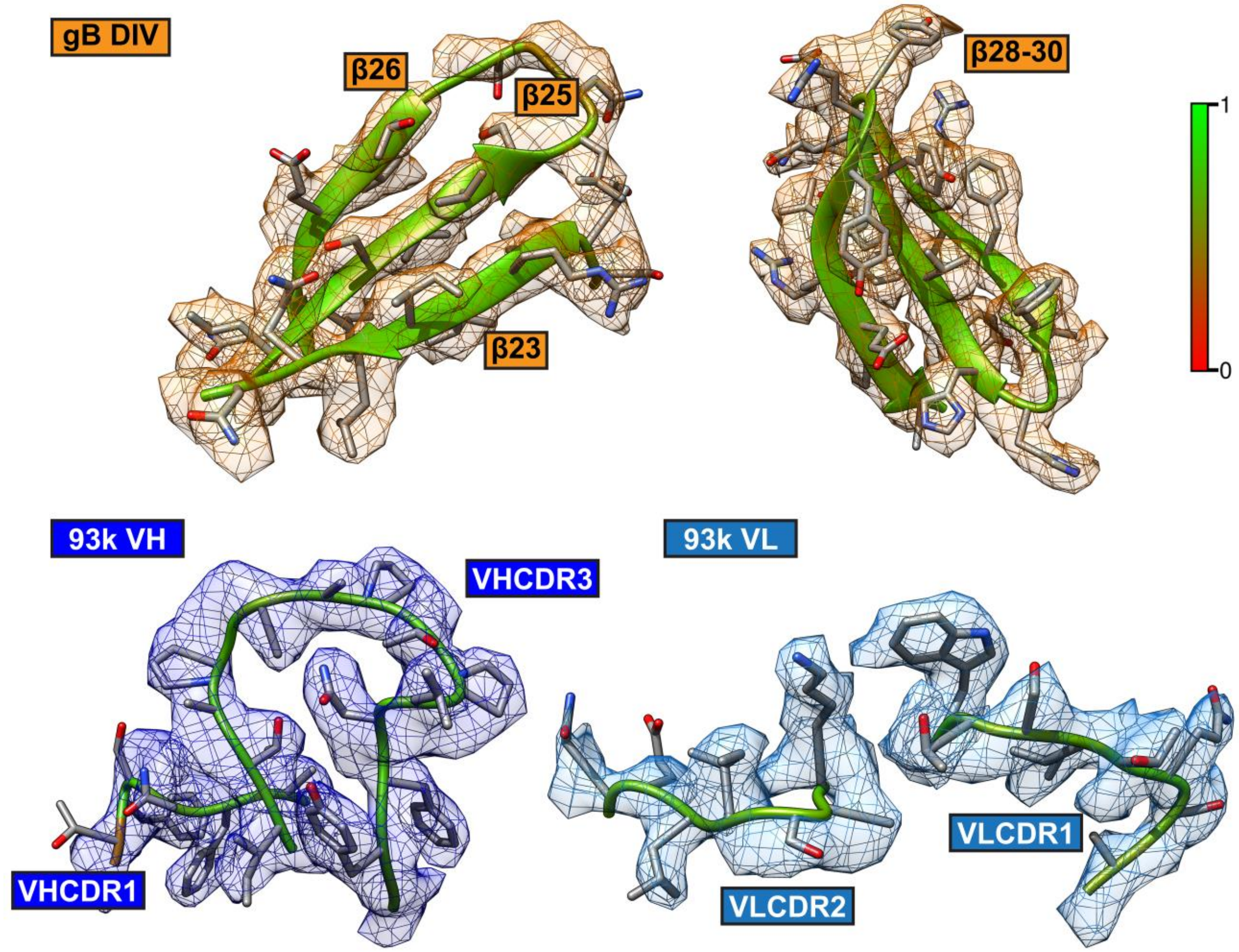

Supplementary Figure 3. Amino acid side chain resolvability at the gB-93k interface in the 2.8Å cryo-EM map. MapQ [3] was used to calculate Q-scores (0 to 1; low to high) for the side chain resolvability. Segmentation and ribbon representations with the amino acid side chains of VZV gB β strands 23, 25, 26 and 28-30 and the 93k VH CDR1, VH CDR3, VL CDR1 and VL CDR2, which form the gB-93k interface. Orientations of the representations are the same as those in Fig. 1G. The scale represents Q-score values.

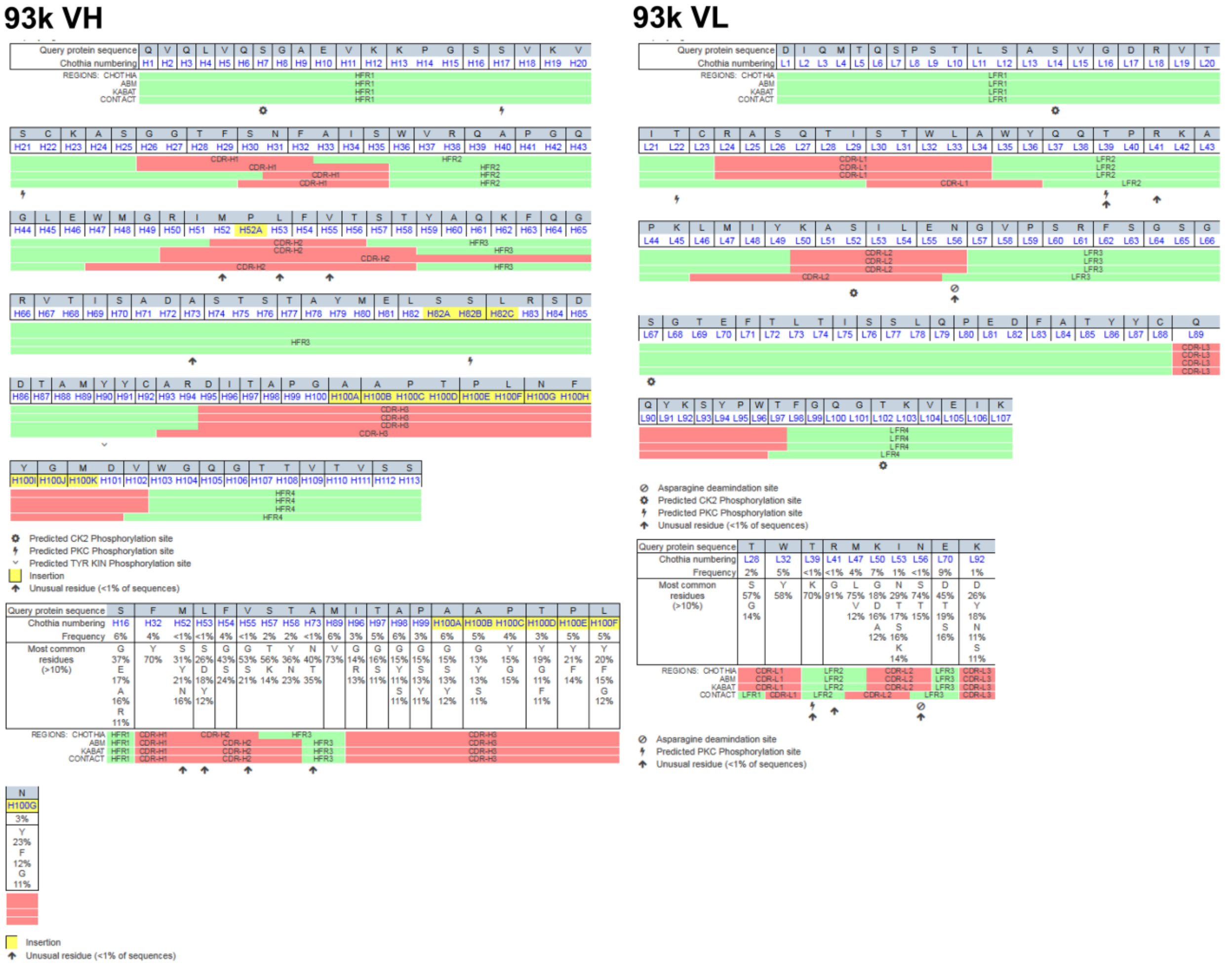

Supplementary Figure 4. Comparison of the mAb 93k VH and VL sequences with all other human monoclonal antibodies. Annotation of the mAb 93k VH and VL chains using the abYsis server (http://www.abysis.org/) to determine the frequency and location of amino acid insertions in the VH or VL chains, and the quantities of unusual amino acids in the CDR regions. Comparison of the mAb 93k VH and VL sequences with all other human monoclonal antibodies (94,157 chains, *Homo sapiens*) revealed three insertion sites in the VH chain (Chothia numbering; H52A, H82A-C and H100A-K) but none in the VL chain. The largest insertion site was within the VHCDR3 and consisted of 11 amino acids not commonly found in human mAbs.

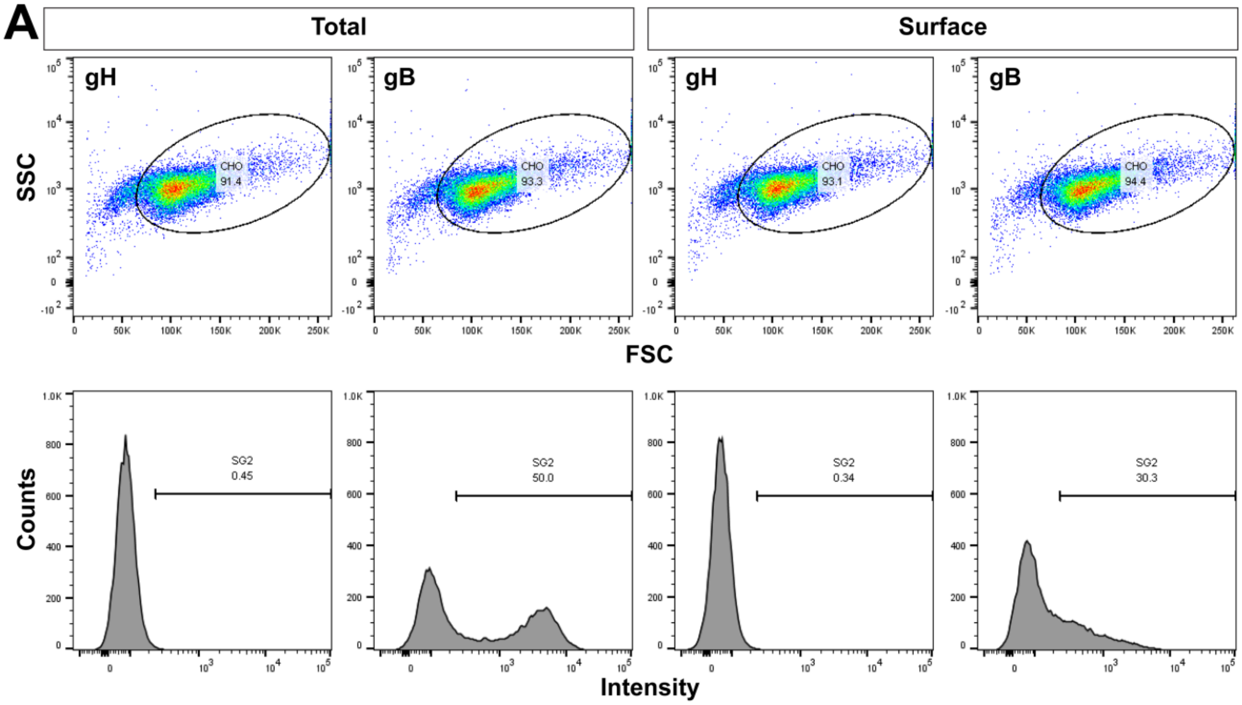
A
Total
Surface
gH
gB
gH
gB
SSC
FSC
CHO
91.4
CHO
93.3
CHO
93.1
CHO
94.4
Counts
Intensity
SG2
0.45
SG2
50.0
SG2
0.34
SG2
30.3

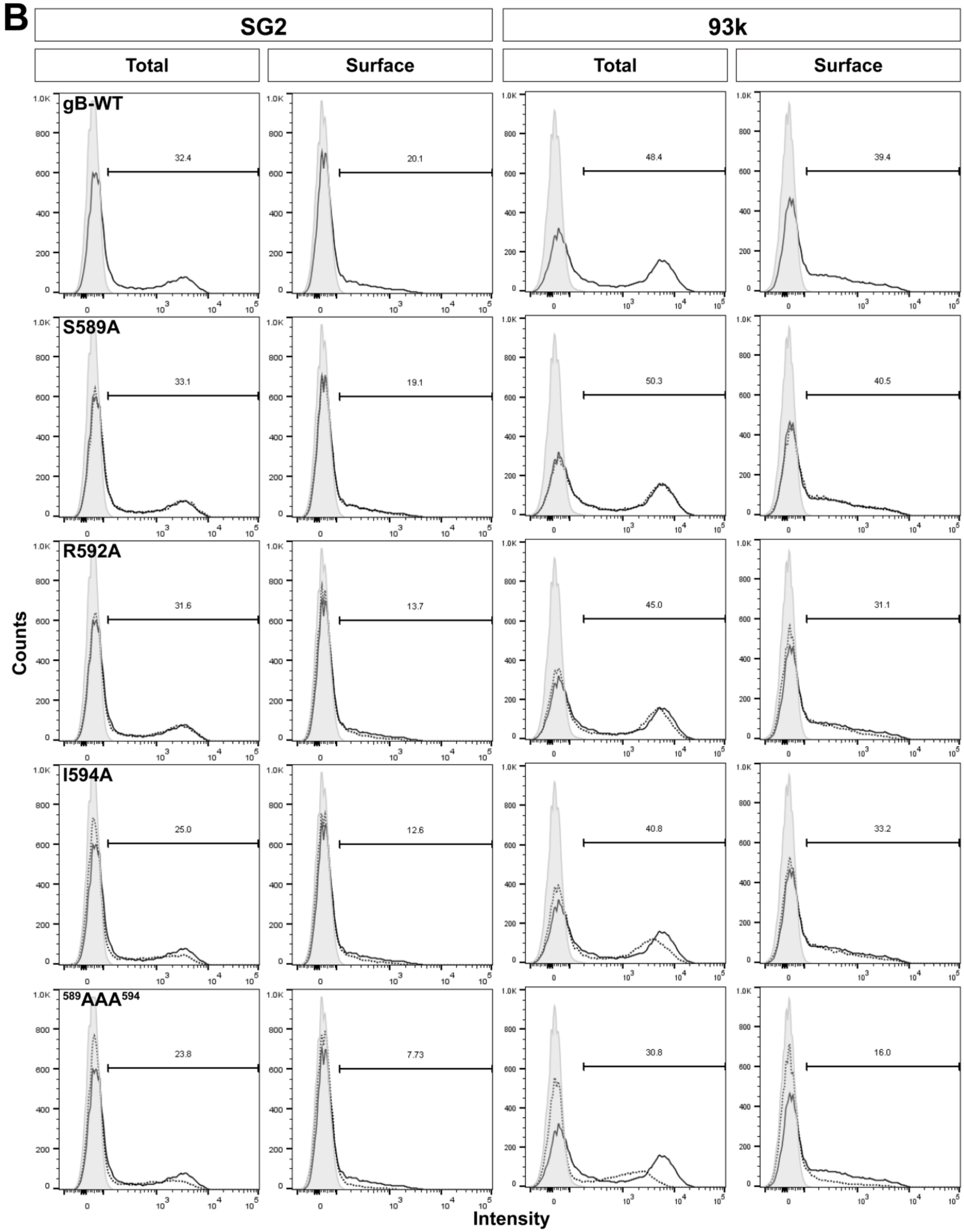
B
SG2
93k
Total
Surface
Total
Surface
gB-WT
S589A
R592A
I594A
589AAA594
32.4
20.1
48.4
39.4
33.1
19.1
50.3
40.5
31.6
13.7
45.0
31.1
25.0
12.6
40.8
33.2
23.8
7.73
30.8
16.0
Counts
Intensity

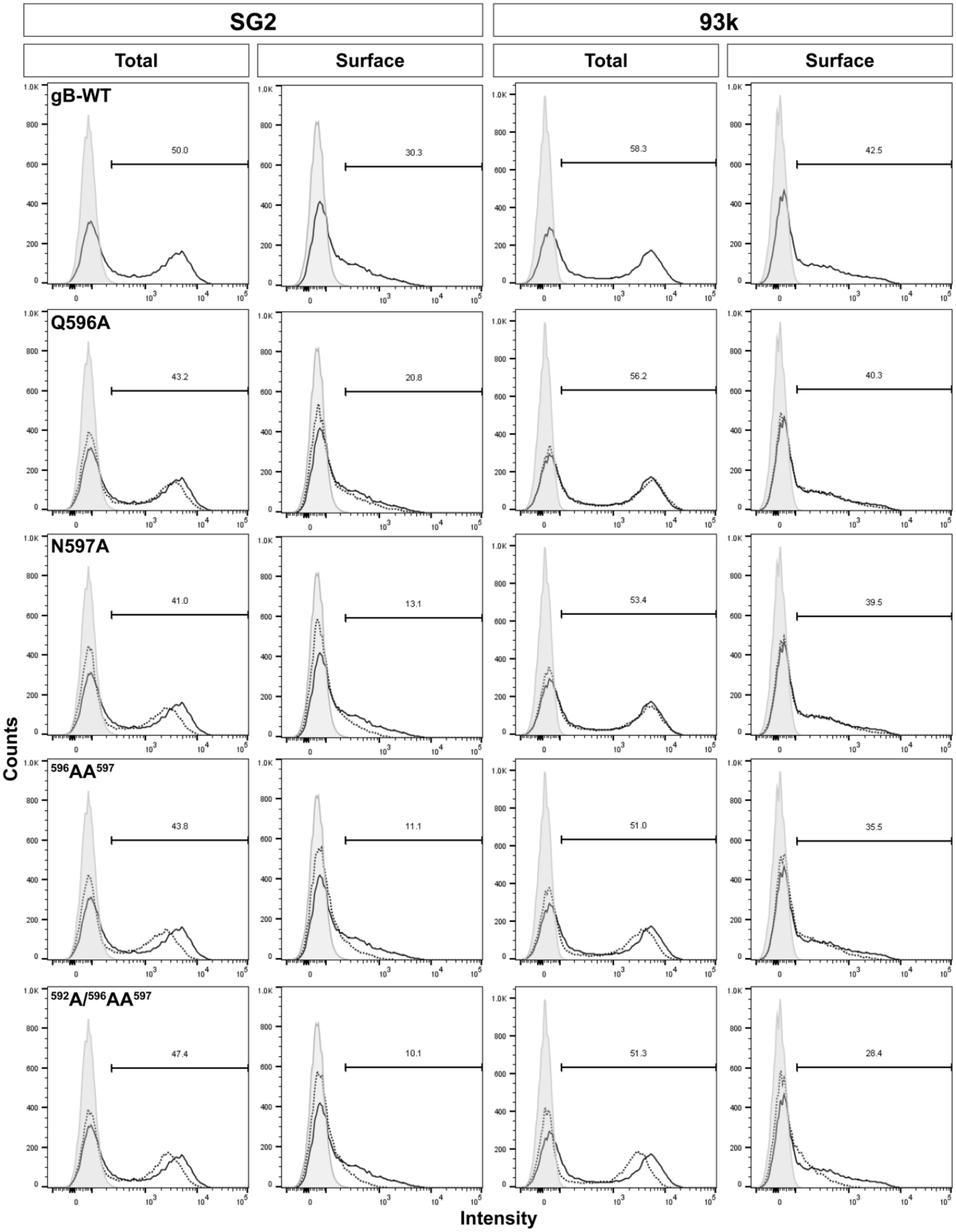

SG2
93k
Total
Surface
Total
Surface
gB-WT
Q596A
N597A
596AA597
592A/596AA597
50.0
30.3
58.3
42.5
43.2
20.8
56.2
40.3
41.0
13.1
53.4
39.5
43.8
11.1
51.0
35.5
47.4
10.1
51.3
28.4
Counts
Intensity

Supplementary Figure 5. The detection of VZV gB β23 mutants on transfected CHO cells by mAbs SG2 and 93k using flow cytometry. A – Gating strategy for the quantification of gB levels using mAbs SG2 and 93k in flow cytometry. The representative samples are for total and surface staining of CHO cells transfected with gH or gB and detected with the anti-gB mAb SG2. The top panels represent examples of CHO cells gated using forward scatter (FSC) and side scatter (SSC). The lower panels represent the histograms from the gated CHO cells and show the specificity of the SG2 mAb for VZV gB. B - Histograms for total and surface stained gB for wild type (WT-gB) and the gB β23 mutants S589, R592, I594, ^{589}AAA^{594}, Q596A, N597A, ^{596}AA^{597} and ^{592}A/^{596}AA^{597} are presented with fluorescence intensity along the abscissa and frequency along the ordinate. In all histograms the negative control (gH) is shaded grey, the positive control (WT-gB) a solid line and each of the mutants is a dotted line. Numbers above the gates (|---|) are the percentage of positive events for either surface stained or total gB as determined by either SG2 or 93k staining.

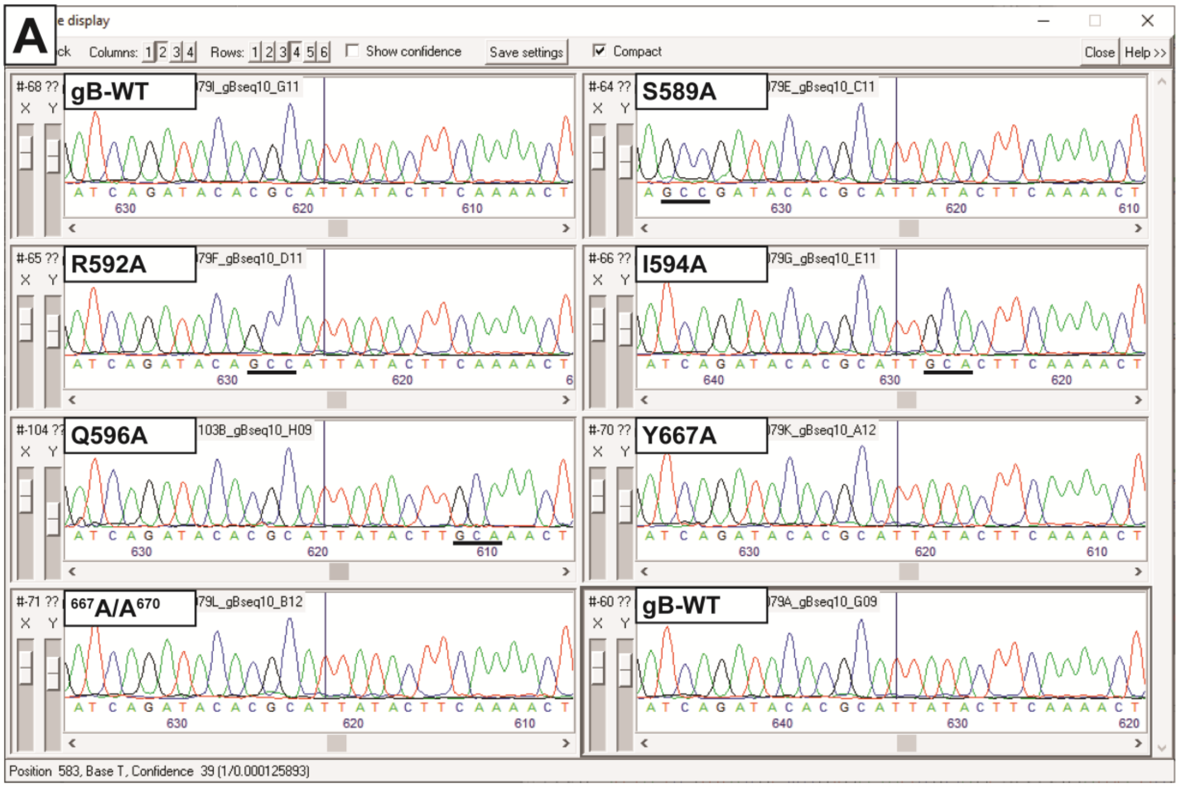

ATCAGATACACGCATTATACTTCAAAACT

S D T R I I L Q N

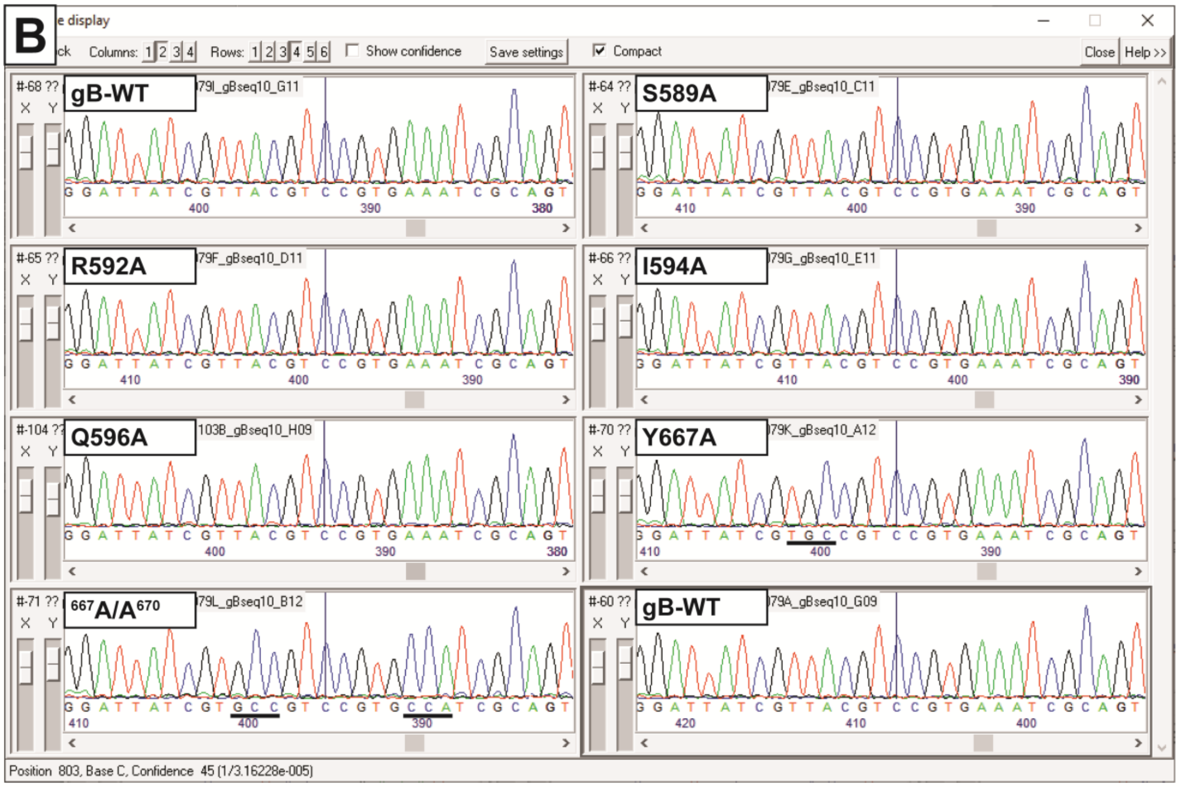

GGATTATCGTTACGTCCGTGAAATCGCAGT

D Y R Y V R E I A

Supplementary Figure 6. Sanger sequencing of VZV gB DIV mutant virus stocks. PCR products generated from ORF31[gB] of virus stocks were sequenced to determine that only the expected mutations were present for each of the VZV gB DIV mutants generated from the transfection of MeWo cells with BACs. Electropherograms in the regions for the β23 (B; S589A, R592A, I594A and Q596A) and β30 (C; Y667 and ^{667}A/A^{670}) mutants are shown with the codon for each of the alanine substitutions underlined. The coding DNA sequence and translated amino acids for gB-WT are provided under each panel with the substituted amino acids highlighted in red.

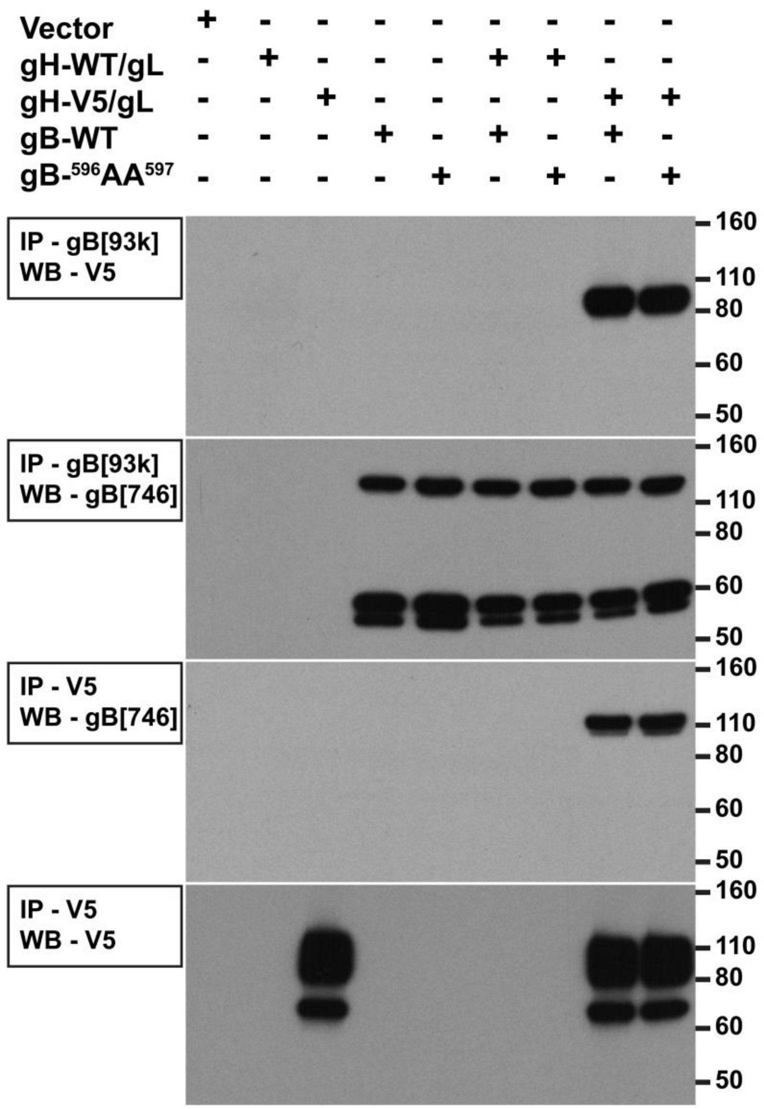

Supplementary Figure 7. VZV gB stably interacts with VZV gH-gL. Western blots (WB) of gB or gH-V5 immunoprecipitated (IP) using mAb 93k or anti-V5 agarose beads from CHO cells transfected with combinations (key above the blots) of gH-WT, gH-V5, gL, gB-WT and the VZV inactivating mutant, gB-^{596}AA^{597}. Western blots were performed using anti-gB Ab 746-868 or a mAb to V5. Numbers to the right of the blots are the values for molecular weight standards (kDa).

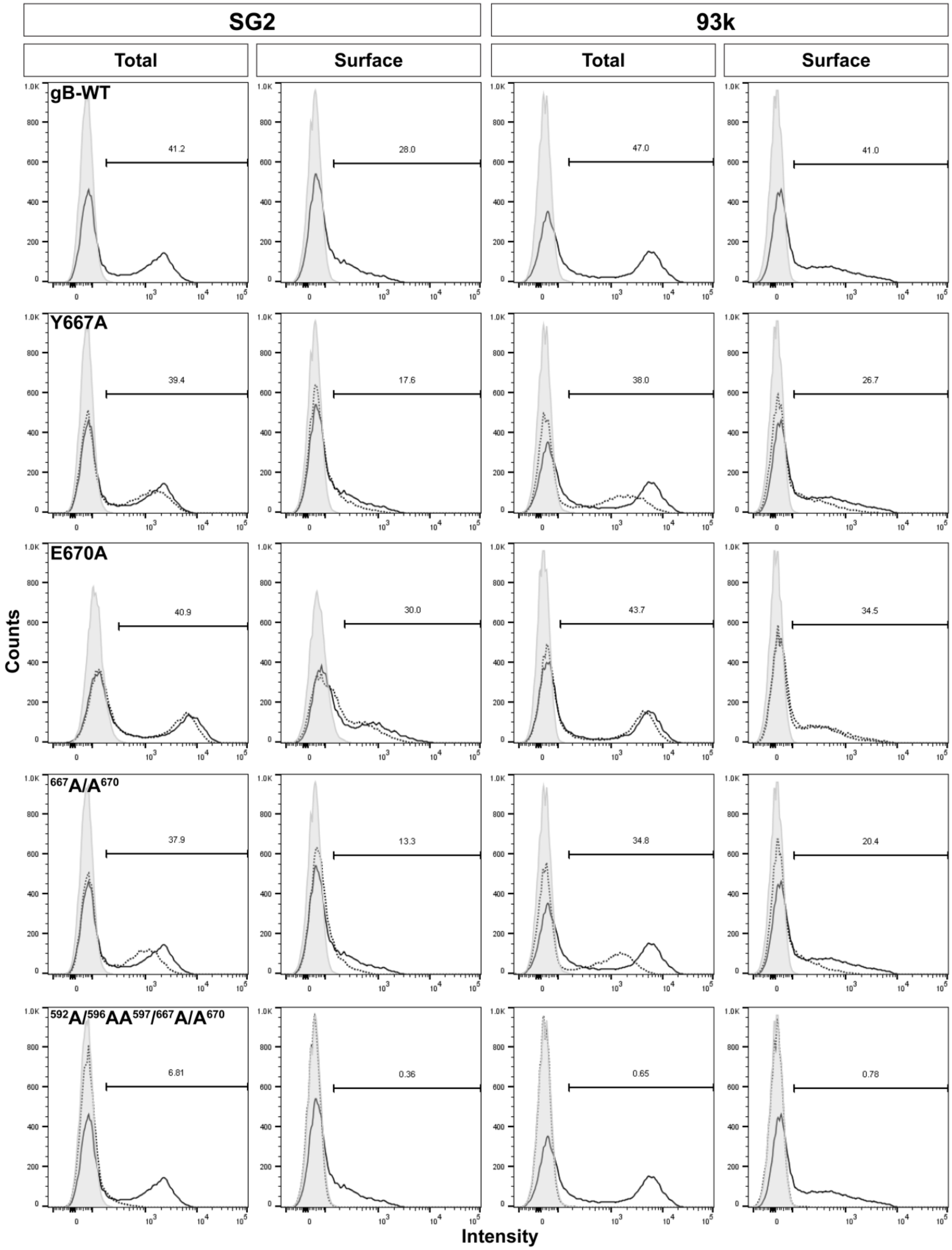

SG2
93k
Total
Surface
Total
Surface
gB-WT
Y667A
E670A
667A/A670
592A/596AA597/667A/A670
Counts
Intensity
41.2
28.0
47.0
41.0
39.4
17.6
38.0
26.7
40.9
30.0
43.7
34.5
37.9
13.3
34.8
20.4
6.81
0.36
0.65
0.78

Supplementary Figure 8. The detection of VZV gB β30 mutants on transfected CHO cells by mAbs SG2 and 93k using flow cytometry. Histograms for total and surface stained gB for wild type (WT-gB) and the gB β30 mutants Y667A, E670A, ^{667}A/A^{670} and ^{592}A/^{596}AA^{597}/^{667}A/A^{670} are presented with fluorescence intensity along the abscissa and frequency along the ordinate. In all of the histograms the negative control (gH) is shaded grey, the positive control (WT-gB) a solid line and each of the mutants a dotted line. Numbers above the gates (|---|) are the percentage of positive events for either surface stained or total gB as determined by either SG2 or 93k staining.

Supplementary Table 1. Cryo-EM data collection, refinement, and validation statistics for the 2.8Å structure of native, full-length VZV gB in complex with Fab fragments from human mAb 93k.

**VZV gB-93k Fab: EMDB- 21247; PDB 6VN1**	
**Data collection and processing**	Titan Krios (FEI; TEM1)
Magnification	130,000X
Voltage (kV)	300
Energy filter slit width (eV)	20
Detector	K2 Summit (Gatan)
Defocus range (μm)	1.5 to 2.0
Pixel size (Å)	1.06
Exposure time (s)	12
Frames	60
Electron dose (e^-/s)	7.5
Electron exposure rate (e^-/Å^2/s)	1.335
Total electron exposure (e^-/ Å^2)	16.02
Symmetry imposed	3
Micrographs collected (no.)	11,283
Final particle images (no.)	856,068
Map resolution (Å)	2.8
FSC threshold	0.143
Map resolution range (Å)	2 to >4
**Refinement**	
Initial model used (PDB code)	6VLK[A]
Model resolution (Å)	2.8
FSC threshold	0.143
Model resolution range (Å)	2 to 4
Model composition	
Chains	12
Non-hydrogen atoms	19,563
Protein residues	2,454
**Validation**	
B factors (Å^2)	
Protein (min/max/mean)	4.8/93.2/23.1
R.M.S. deviations	
Bond lengths (Å)	0.006
Bond angles (°)	0.616
Validation	
MolProbity score	1.85
Clashscore	7
Poor rotamers (%)	0
Ramachandran plot	
Favored (%)	94
Allowed (%)	6
Disallowed (%)	0

[A] X-ray crystallography data for VZV gB

Supplementary Table 2. Amino acid residues and color code for each domain in VZV gB.

Residues	Domain	Color
115-136	IV	Orange
137-147	Linker: II-IV	Hot Pink
148-159	II	Green
160-368	I	Cyan
369-464	II	Green
465-502	II?	
503-510	Linker: II-III	Hot Pink
511-569	III	Yellow
570-681	IV	Orange
682-736	V	Red

Supplementary Table 3. Conserved cysteine bonds in herpesvirus gB orthologues.

**Domain Location**	**Cysteine Residue Locations in gB**				
	**VZV**	**HSV**	**PRV**	**HCMV**	**EBV**
DIV	122-584	116-573	129-603	94-551	51-528
DII/IV linker to DIII	139-540	133-529	146-559	111-507	68-484
DI	213-277	207-271	220-284	185-250	141-206
DII	369-417	364-412	377-426	344-391	295-342
DIV	608-645	596-633	625-661	574-611	551-588

Supplementary Table 4. Q-score values for the amino acids that comprise the gB-93k interface.

Protein	Subdomain	Amino acid[A]	Q-score[B]	
			Cα	Side Chain
VZV gB	DIV N-terminus	T115	0.84	0.66
		K116	0.85	0.72
	DIV β23	S589	0.64	0.48
		R592	0.77	0.68
		I593	0.70	0.74
		I594	0.72	0.74
		L595	0.76	0.71
		Q596	0.77	0.69
		N597	0.73	0.71
	DIV β25	S615	0.77	0.68
		V617	0.80	0.71
		L619	0.70	0.66
	DIV β28	F655	0.74	0.69
	DIV β29	H658	0.73	0.73
	DIV β30	Y667	0.72	0.65
		E670	0.67	0.62
93k VH	CDR2	N31	0.71	0.63
	CDR3	I100	0.80	0.71
		T101	0.71	0.72
		A102	0.80	0.66
		P103	0.78	0.70
		G104	0.82	0.75
		A105	0.72	0.64
		A106	0.82	0.68
		P107	0.67	0.68
		T108	0.73	0.69
		P109	0.74	0.71
		L110	0.76	0.71
		N111	0.75	0.72
		Y113	0.74	0.72
93k VL	CDR1	W32	0.71	0.73
	CDR2	Y49	0.75	0.72
		I53	0.77	0.67
		E55	0.82	0.65
		N56	0.63	0.57

[A]Molecular interactions between the gB and 93k amino acids were calculated using UCSF Chimera[4].
[B]Q-scores for Cα and the amino acid side chains was calculated using MapQ [3].

Supplementary Table 5. Key reagents and resources.

REAGENT or RESOURCE	SOURCE	IDENTIFIER
**Antibodies**		
Human mAb 93k	This paper	
Mouse mAb SG2-2E6	GeneTex	GTX38718
Mouse mAb 206	[5]	
Rabbit polyclonal antibody 746-868	[6]	
Mouse mAb IE62	EMD Millipore	MAB8616
Mouse anti-V5 tag	Bio-Rad	MCA1360
VZV mouse mixed mAb	Meridian Life Sciences	C05108MA
Biotinylated goat anti-mouse IgG (H+L)	Vector Labs. Inc.	BA-9200
Donkey anti-mouse Alexa Fluor 555	Life Technologies	A31570
Goat anti-human Alexa Fluor 488	Life Technologies	A11013
ECL™ Sheep anti-mouse IgG, Horseradish peroxidase linked whole antibody	GE Healthcare UK Ltd	NA931V
ECL™ Donkey anti-rabbit IgG, Horseradish peroxidase linked whole antibody	GE Healthcare UK Ltd	NA934V
ECL™ Sheep anti-human IgG, Horseradish peroxidase linked whole antibody	GE Healthcare UK Ltd	NA933V
**Bacterial and Virus Strains**		
pOka BAC derived (pPOKA-DX)	[7]	
GS1783	[8]	
pOka-TK-GFP	[9]	
pOka-TK-GFP gB-TEVV5	This paper	
pOka-TK-GFP gB-TEVV5 gB[S110A]	This paper	
pOka-TK-GFP gB-TEVV5 gB[Q111A]	This paper	
pOka-TK-GFP gB-TEVV5 gB[D112A]	This paper	
pOka-TK-GFP gB-TEVV5 gB[^{109}AAAA^{112}]	This paper	
pOka-TK-GFP gB-TEVV5 gB[S589A]	This paper	
pOka-TK-GFP gB-TEVV5 gB[R592A]	This paper	
pOka-TK-GFP gB-TEVV5 gB[I594A]	This paper	
pOka-TK-GFP gB-TEVV5 gB[Q596A]	This paper	
pOka-TK-GFP gB-TEVV5 gB[Y667A]	This paper	
pOka-TK-GFP gB-TEVV5 gB[^{667}A/A^{670}]	This paper	
**Chemicals, Peptides, and Recombinant Proteins**		
Anti-V5 Agarose Affinity Gel	Sigma	A7345-1ML
Bovine Serum Albumin (IgG Free, Protease Free)	Jackson ImmunoResearch	001-000-162
Minimal essential medium	Corning cellgro	10-010-CV
F-12K nutrient mixture Kaighn's modification	Invitrogen	21127-022
Optimem +Glutamax	Gibco	51985-034
Fetal bovine serum	Gibco	26140-079

Penicillin /Streptomycin	Gibco	15140-122
Amphotericin B	Corning cellgro	30-003-CF
Nonessential amino acids	Corning cellgro	25-025-CI
Puromycin	Invitrogen	A11138-03
BstZ171	New England BioLabs Inc.	R3594
NaeI	New England BioLabs Inc.	R0190L
HindIII	New England BioLabs Inc.	R0104L
AgeI	New England BioLabs Inc.	R0552L
SpeI	New England BioLabs Inc.	R0133L
NotI	New England BioLabs Inc.	R0189L
KpnI	New England BioLabs Inc.	R0142L
XmaI	New England BioLabs Inc.	R0180L
NdeI	New England BioLabs Inc.	R0111L
AccuPrime™ *Pfx* DNA Polymerase	Invitrogen	12344024
KOD Extreme™ Hot Start DNA Polymerase	EMD Millipore	71975-3
Lipofectamine® 2000	Invitrogen	11668-019
Ampicillin	Sigma	A9518-100G
Kanamycin	Sigma	K4378
LB (Miller's) Agar	Growcells	MBPE-3060
LB (Miller's) Broth	Growcells	MBPE-1050
PBS (Phosphate buffered saline)	Corning cellgro	21-040-CV
DPBS (Dulbecco's phosphate buffered saline)	Corning cellgro	21-030-CV
Tris Base	Fisher Scientific	BP152-5
Sodium chloride	Fisher Scientific	S271-10
Potassium chloride	Fisher Scientific	BP366-500
Magnesium chloride	Fisher Scientific	M33-500
Sodium deoxycholate	ICN Biomedical Inc.	804312
IGEPAL® CA-630	Sigma	I3021-100ML
Triton X-100	Sigma	T-9284
Tobacco etch virus protease	In house	
Tris buffered saline (TBS; pH7.4)	Scy Tek	TBS500
Amphipol 8-35	Anatrace	A835
Lauroylsarcosine	Sigma	L9150
Bio-Beads™ SM-2	Bio-Rad	152-3920
Phosphotungstic acid	Ted Pella	19402
EDTA	Sigma	E9884
Sucrose	Sigma	S7903-1KG

L(+)-glutamic acid monosodium salt monohydrate	Sigma	G1626-100G
Paraformaldehyde (4%) in PBS	Boston Bioproducts	K06J101
BD Cytofix/Cytoperm™ Plus	BD Biosciences	555028
Hoechst 33342	ThermoFisher Scientific	H-3570
Native PAGE™ Sample Buffer	Novex	BN20032
Native PAGE™ running buffer 20X	Novex	BN2001
Native PAGE™ 20X cathode buffer additive	Novex	BN2002
Native PAGE™ 3-12% Bis Tris Gel	Novex	BN2011BX10
Laemmli Sample Buffer 2X	Bio-Rad	161-0737
2-mercaptoethanol	Sigma	M7522-100ML
Mini Protean® TGX™ Gels 4-20%	Bio-Rad	456-1094
NativeMark™ Protein Std.	Invitrogen	57030
Novex® Sharp Pre-Stained Protein Standards	Invitrogen	57318
Dimethyl pimelimidate dihydrochloride	Sigma	D8388
Protein A Plus UltraLink® Resin	Thermo Scientific	53142
Fluoromount-G®	SouthernBiotech	0100-01
Boric acid	Sigma	B-0252
Ethanolamine	Sigma	398136-500ML
Ethane	Airgas	ET R80
L-(+)-Arabinose	Sigma	A3256-100G
Agarose LE	AccuFlow	EK2808
Membrane permeable coelenterazine-H	Nanolight Technology	3012-10
Fast Red TR Salt hemi (zinc chloride)	Sigma	368881-25G
Naphthol AS-MX phosphate	Sigma	N4875-500MG
Alkaline phosphatase-conjugated Streptavidin	Jackson ImmunoResearch	016-050-084
**Experimental Models: Cell Lines**		
MeWo	ATCC	HTB-65
CHO-DSP1	[10]	
Mel-DSP2	[10]	
**Oligonucleotides (All sequences are 5' to 3')**		
**TEVV5**		
gB-AgeI<br>CTTTTTTGCGTACCGGTACGTGC	This study	Elim Bio
gB931<br>[PHOS]CACCCCCGTTACATTCTCGGTGCG	This study	Elim Bio
gB-V5<br>[PHOS]GGTAAGCCTATCCCTAACCCTCTCCTCGGTCTCGA TTCTACGTAAATAGCCAGGGGGTTT	This study	Elim Bio
M13R<br>CACCCCCGTTACATTCTCGGTGCG	Invitrogen	

gB-Cterm-S-tag<br>[PHOS]GCTGTCCATGTGCTGGCGTTCGAATTTAGCAGCAGCGGTTTCTTTCACCCCCGTTACATTCTCGG	This study	Elim Bio
gB-link_TEV_link<br>[PHOS]*GGCGGCGGGGGCGGG*GAGAATCTTTATTTTCAGG*GCGGGGGCG*GGGGTAAGCCTATCCCTAACCC	This study	Elim Bio
ΔS-tag-sense<br>[PHOS]GGCGGCGGGGGCGGGGAGATTC	This study	Elim Bio
ΔS-tag-antisense<br>[PHOS]CACCCCCGTTACATTCTCGGTG	This study	Elim Bio
[31]F56625-56645<br>AGGTATAGGCAGTTCCCACGG	[11]	Elim Bio
[31]R59697-59717<br>TTTCATTGAGACTTGAAGCGC	[11]	Elim Bio
**gB SRI 589/592/594**		
pCAGGs-gB-XmaI-sense<br>AGGAAGCCCGGGCTATTATTAACC	This study	Elim Bio
S589A-antisense<br>[PHOS]TGTATCGGCTCCCAGTTCTGGACAATTAG	This study	Elim Bio
S589A-sense<br>[PHOS]CGCATTATACTTCAAAACTC	This study	Elim Bio
pCAGGs-gB-AgeI-antisense<br>GCACGTACCGGTACGCAAAAAAGG	This study	Elim Bio
R592A-antisense<br>[PHOS]TATAATGGCTGTATCTGATCCCAGTTCTG	This study	Elim Bio
pCAGGs-gB-3617-sense<br>[PHOS]CTTCAAAACTCTATGAGGGTA	This study	Elim Bio
I594A-antisense<br>[PHOS]TTGAAGTGCAATGCGTGTATCTGATCCCA	This study	Elim Bio
589AAA594-antisense<br>[PHOS]TATCGGCTCCCAGTTCTGGACAATTAGAAAC	This study	Elim Bio
589AAA594-sense<br>[PHOS]CAGCCATTGCACTTCAAAACTCTATGAGGGTATC	This study	Elim Bio
**gB Q596A/N597A**		
Q596A-antisense<br>[PHOS]TGCAAGTATAATGCGTGTATCTG	This study	Elim Bio
pCAGGs-gB-3623-sense<br>[PHOS]AACTCTATGAGGGTATCTGGTAG	This study	Elim Bio
pCAGGs-gB-3622-antisense<br>[PHOS]TTGAAGTATAATGCGTGTATCTG	This study	Elim Bio
N597A-sense<br>[PHOS]GCGTCTATGAGGGTATCTGGTAG	This study	Elim Bio
R592A-Q596A-antisense<br>[PHOS]TGCAAGTATAATGGCTGTATCTG	This study	Elim Bio
pCAGGs-gB-KpnI-sense<br>AACGGGAATTGGTACCCTATCAGCA	This study	Elim Bio

pCAGGs-gB-NotI-BstZ171<br>TTAGCGGCCGCGAATTCGCCCTTGTATACACCCTAATGCAG CGGCTGG	This study	Elim Bio
**gB-Y667A/E670A**		
pCAGGs-gB-MluI-sense<br>GGTAGTACTACGCGTTGTTATAGC	This study	Elim Bio
Y667A-antisense<br>[PHOS]GGCACGATAATCCTCATAATATACG	This study	Elim Bio
V668-sense<br>[PHOS]GTCCGTGAAATCGCAGTCCATGATG	This study	Elim Bio
Y667-antisense<br>[PHOS]GTAACGATAATCCTCATAATATACG	This study	Elim Bio
E670A-sense<br>[PHOS]GTCCGTGCCATCGCAGTCCATGATG	This study	Elim Bio
**Recombinant DNA**		
pRS5a-93k	This study	
pCAGGs-VZVgB	[12]	
pME18s	[12]	
pME18s-gH[TL]	[12]	
pME18s-gH[V5]	[9]	
pCDNA3.1(+)	Invitrogen	V79020
pCDNA3.1-gL	[13]	
pCAGGs-gB[S589A]	This study	
pCAGGs-gB[R592A]	This study	
pCAGGs-gB[I594A]	This study	
pCAGGs-gB[^{589}AAA^{594}]	This study	
pCAGGs-gB[Q596A]	This study	
pCAGGs-gB[N597A]	This study	
pCAGGs-gB[^{596}AA^{597}]	This study	
pCAGGs-gB[^{592}A/^{596}AA^{597}]	This study	
pCAGGs-gB[Y667A]	This study	
pCAGGs-gB[E670A]	This study	
pCAGGs-gB[^{667}A/A^{670}]	This study	
pCAGGs-gB[^{592}A/^{596}AA^{597}/^{667}A/A^{670}]	This study	
gB-Kan	[11]	
gB-Kan-TEVV5	This study	
pPOKA-TK-GFP-BAC-DX ΔORF31	[9]	
pPOKA-TK-GFP gB-TEVV5 gB[S589A]	This study	
pPOKA-TK-GFP gB-TEVV5 gB[R592A]	This study	
pPOKA-TK-GFP gB-TEVV5 gB[I594A]	This study	
pPOKA-TK-GFP gB-TEVV5 gB[Q596A]	This study	

pPOKA-TK-GFP gB-TEVV5 gB[N597A]	This study	
pPOKA-TK-GFP gB-TEVV5 gB[^{596}AA^{597}]	This study	
pPOKA-TK-GFP gB-TEVV5 gB[^{592}A/^{596}AA^{597}]	This study	
pPOKA-TK-GFP gB-TEVV5 gB[Y667A]	This study	
pPOKA-TK-GFP gB-TEVV5 gB[E670A]	This study	
pPOKA-TK-GFP gB-TEVV5 gB[^{667}A/A^{670}]	This study	
pPOKA-TK-GFP gB-TEVV5 gB[^{592}A/^{596}AA^{597}/^{667}A/A^{670}]	This study	
**Software and Algorithms**		
SerialEM v3.7	[14]	http://bio3d.colorado.edu/SerialEM/
Relion v3.0	[15,16]	https://bitbucket.org/scheres/relion-3.0_beta/src/master/
ResMap v1.95	[1]	https://sourceforge.net/projects/resmap-latest/
UCSF Chimera v1.13.1	[4]	http://www.cgl.ucsf.edu/chimera/
MapQ v1.5.4	[3]	https://cryoem.slac.stanford.edu/ncmi/resources/software/mapq
Phenix v1.17.1-3660	[2]	https://www.phenix-online.org/
WinCoot v0.8.9.2	[17]	http://bernhardcl.github.io/coot/
FlowJo CE 7.5.110.7	TreeStar	
FlowJo v10.6.2	TreeStar	
Prism 8 v8.4.1	GraphPad Software, Inc.	
Staden Pregap v1.5 Gap v4.10	[18]	http://staden.sourceforge.net
GeneDoc v2.7	Nicholas and Nicholas, 1997	https://genedoc.software.informer.com/download/
BZ-X Viewer v1.3.0.6	Keyence	
BZ-X Analyzer v1.3.0.3	Keyence	
AxioVision v4.8	Zeiss	
FiJi (ImageJ 1.52i)	NIH, USA	http://imagej.nih.gov/ij
Illustrator CS6	Adobe	
Photoshop CS6	Adobe	

**Other**		
NativePAGE™ 2-12% Bis-Tris Gel	Invitrogen	BN2011BX10
Mini-PROTEAN® TGX™ Gels	Bio-Rad	456-1094
Superose-6 Increase 3.2x300mm	Sigma	GE29-0915-98
Ultrathin carbon film on lacey carbon support 400M Cu	Ted Pella	01824
Quantifoil® R 1.2/1.3 Au 300 mesh grids	Quantifoil	
Leica EM GP	Leica	
Titan Krios	FEI	
Amicon Ultra-4 Centrifugal Filter Units 100kDa	Millipore	UFC810024
Amicon Ultra-4 Centrifugal Filter Units 10kDa	Millipore	UFC801024
QIAquick® Gel Extraction Kit	Qiagen	28706
QIAquick® Nucleotide Removal Kit	Qiagen	28304
QIAprep® Spin Miniprep Kit	Qiagen	27106
QIAGEN® Large-Construct Kit	Qiagen	12462
Immobilon®-P	Merck Millipore Ltd.	IPVH00010
Optical bottom 96-well black sided culture plates	Thermo Scientific	165305
FACSCalibur flow cytometer	Becton Dickenson	
Synergy H1 Multi-mode Reader	Biotek	
Nunclon™ Delta Surface 12-well plates	Thermo Scientific	150628
Cell Culture 6-well plates	Corning	3506
Microscope cover glass 18mm No. 1	Fisher Scientific	12-545-100

References.

1 Kucukelbir, A., Sigworth, F. J. & Tagare, H. D. Quantifying the local resolution of cryo-EM density maps. *Nat Methods* **11**, 63-65, (2014).

2 Adams, P. D. *et al.* PHENIX: a comprehensive Python-based system for macromolecular structure solution. *Acta Crystallogr D Biol Crystallogr* **66**, 213-221, (2010).

3 Pintilie, G. *et al.* Measurement of atom resolvability in cryo-EM maps with Q-scores. *Nat Methods* **17**, 328-334, (2020).

4 Pettersen, E. F. *et al.* UCSF Chimera--a visualization system for exploratory research and analysis. *J Comput Chem* **25**, 1605-1612, (2004).

5 Montalvo, E. A. & Grose, C. Neutralization epitope of varicella zoster virus on native viral glycoprotein gp118 (VZV glycoprotein gpIII). *Virology* **149**, 230-241, (1986).

6 Oliver, S. L. *et al.* Mutagenesis of varicella-zoster virus glycoprotein B: putative fusion loop residues are essential for viral replication, and the furin cleavage motif contributes to pathogenesis in skin tissue in vivo. *J Virol* **83**, 7495-7506, (2009).

7 Tischer, B. K. *et al.* A self-excisable infectious bacterial artificial chromosome clone of varicella-zoster virus allows analysis of the essential tegument protein encoded by ORF9. *J Virol* **81**, 13200-13208, (2007).

8 Tischer, B. K., Smith, G. A. & Osterrieder, N. En passant mutagenesis: a two step markerless red recombination system. *Methods Mol Biol* **634**, 421-430, (2010).

9 Yang, E., Arvin, A. M. & Oliver, S. L. The cytoplasmic domain of varicella-zoster virus glycoprotein H regulates syncytia formation and skin pathogenesis. *PLoS Pathog* **10**, e1004173, (2014).

10 Yang, E., Arvin, A. M. & Oliver, S. L. Role for the alphaV Integrin Subunit in Varicella-Zoster Virus-Mediated Fusion and Infection. *J Virol* **90**, 7567-7578, (2016).

11 Oliver, S. L. *et al.* An immunoreceptor tyrosine-based inhibition motif in varicella-zoster virus glycoprotein B regulates cell fusion and skin pathogenesis. *Proc Natl Acad Sci U S A* **110**, 1911-1916, (2013).

12 Suenaga, T. *et al.* Myelin-associated glycoprotein mediates membrane fusion and entry of neurotropic herpesviruses. *Proc Natl Acad Sci U S A* **107**, 866-871, (2010).

13 Vleck, S. E. *et al.* Structure-function analysis of varicella-zoster virus glycoprotein H identifies domain-specific roles for fusion and skin tropism. *Proc Natl Acad Sci U S A* **108**, 18412-18417, (2011).

14 Mastronarde, D. N. Automated electron microscope tomography using robust prediction of specimen movements. *J Struct Biol* **152**, 36-51, (2005).

15 Zivanov, J. *et al.* New tools for automated high-resolution cryo-EM structure determination in RELION-3. *Elife* **7**, (2018).

16 Scheres, S. H. RELION: implementation of a Bayesian approach to cryo-EM structure determination. *J Struct Biol* **180**, 519-530, (2012).

17 Emsley, P., Lohkamp, B., Scott, W. G. & Cowtan, K. Features and development of Coot. *Acta Crystallogr D Biol Crystallogr* **66**, 486-501, (2010).

18 Staden, R. The Staden sequence analysis package. *Mol Biotechnol* **5**, 233-241, (1996).
