# Supplementary material for: A glycoprotein B-neutralizing antibody structure at 2.8 Å uncovers a critical domain for herpesvirus fusion initiation
Source: Nat Commun. 2020 Aug 18;11:4141. doi: 10.1038/s41467-020-17911-0 (PMC7435202; doi:10.1038/s41467-020-17911-0)
Supplement: Supplementary file 3 — Reporting Summary [file 41467_2020_17911_MOESM3_ESM.pdf]

nature research

Corresponding author(s): Stefan L. Oliver

Last updated by author(s): Jul 1, 2020

Reporting Summary

Nature Research wishes to improve the reproducibility of the work that we publish. This form provides structure for consistency and transparency in reporting. For further information on Nature Research policies, see our [Editorial Policies] and the [Editorial Policy Checklist].

Statistics

For all statistical analyses, confirm that the following items are present in the figure legend, table legend, main text, or Methods section.

n/a	Confirmed	
☐	☒	The exact sample size (*n*) for each experimental group/condition, given as a discrete number and unit of measurement
☐	☒	A statement on whether measurements were taken from distinct samples or whether the same sample was measured repeatedly
☐	☒	The statistical test(s) used AND whether they are one- or two-sided<br>*Only common tests should be described solely by name; describe more complex techniques in the Methods section.*
☒	☐	A description of all covariates tested
☐	☒	A description of any assumptions or corrections, such as tests of normality and adjustment for multiple comparisons
☐	☒	A full description of the statistical parameters including central tendency (e.g. means) or other basic estimates (e.g. regression coefficient) AND variation (e.g. standard deviation) or associated estimates of uncertainty (e.g. confidence intervals)
☒	☐	For null hypothesis testing, the test statistic (e.g. *F*, *t*, *r*) with confidence intervals, effect sizes, degrees of freedom and *P* value noted<br>*Give P values as exact values whenever suitable.*
☒	☐	For Bayesian analysis, information on the choice of priors and Markov chain Monte Carlo settings
☒	☐	For hierarchical and complex designs, identification of the appropriate level for tests and full reporting of outcomes
☒	☐	Estimates of effect sizes (e.g. Cohen's *d*, Pearson's *r*), indicating how they were calculated

Our web collection on statistics for biologists contains articles on many of the points above.

Software and code

Policy information about availability of computer code

Data collection	SerialEM v3.7, CellQuest Pro, AxioVision v4.8, FlowJo CE 7.5.110.7, BZ-X Viewer v1.3.0.6
Data analysis	Revion v3.0, Phenix v1.17.1-3660, WinCoot v0.8.9.2, ResMap v1.95, MapQ v1.5.4, Prism 8 v8.4.1, Staden Pregap v1.5 Gap v4.10, GeneDoc v2.7, FiJi (ImageJ v1.52i)

For manuscripts utilizing custom algorithms or software that are central to the research but not yet described in published literature, software must be made available to editors and reviewers. We strongly encourage code deposition in a community repository (e.g. GitHub). See the Nature Research guidelines for submitting code & software for further information.

Data

Policy information about availability of data

All manuscripts must include a data availability statement. This statement should provide the following information, where applicable:

- Accession codes, unique identifiers, or web links for publicly available datasets
- A list of figures that have associated raw data
- A description of any restrictions on data availability

Maps and models have been deposited in the Electron Microscopy Data Bank with accession code 21247 and the Protein Data Bank with accession code 6VN1. The authors declare that the data supporting the findings of this study are available within the paper and its Supplementary Information. Source data are provided with this paper. Each data point corresponding to figures and western blots that describe the results are provided as separate Source Data for Figs. 1A,1B, 5B, 5D-F, 6B and 6D-F. All primary data will be provided by the corresponding author upon reasonable request.

Field-specific reporting

Please select the one below that is the best fit for your research. If you are not sure, read the appropriate sections before making your selection.

☒ Life sciences ☐ Behavioural & social sciences ☐ Ecological, evolutionary & environmental sciences

For a reference copy of the document with all sections, see nature.com/documents/nr-reporting-summary-flat.pdf

Life sciences study design

All studies must disclose on these points even when the disclosure is negative.

Sample size	Sample size calculations were based on our previous published studies. For transfection-based studies, a minimum of two clones were evaluated in quantitative experiments. For VZV infection-based studies a minimum of two experiments were performed with three biological replicates. Sample sizes were sufficient as statistical significance was reached using two-way ANOVA.
Data exclusions	Data were not excluded from the analyses.
Replication	All experiments were performed at minimum in two independent experiments with at least two biological replicates. All attempts at replicating experiments were successful.
Randomization	Randomization was not performed as quantitative data were derived from cell culture-based experiments.
Blinding	The corresponding author planned and performed all quantitative experiments therefore blinding of the data was not performed.

Reporting for specific materials, systems and methods

We require information from authors about some types of materials, experimental systems and methods used in many studies. Here, indicate whether each material, system or method listed is relevant to your study. If you are not sure if a list item applies to your research, read the appropriate section before selecting a response.

Materials & experimental systems

n/a	Involved in the study	
☐	☒	Antibodies
☐	☒	Eukaryotic cell lines
☒	☐	Palaeontology and archaeology
☒	☐	Animals and other organisms
☒	☐	Human research participants
☒	☐	Clinical data
☒	☐	Dual use research of concern

Methods

n/a	Involved in the study	
☒	☐	ChIP-seq
☐	☒	Flow cytometry
☒	☐	MRI-based neuroimaging

Antibodies

Antibodies used	Mouse mAb anti-gB SG2-2E6 (GeneTex GTX38718) used in VZV neutralization and fusion inhibition assays (10ug/ml), flow cytometry (1:100), immunoprecipitation (20ug/30ul beads); human mAb anti-gB 93k (purified in house 1mg/ml) used in VZV neutralization and fusion inhibition assays (10ugml), flow cytometry (1:100), immunoprecipitation (20ug/30ul beads), western blot (1:5,000); mouse mAb anti-gH 206 (1mg/ml, Montalvo & Grose, 1986. Virology 149, 230-241) used in VZV neutralization and fusion inhibition assays; mouse mAb anti-IE62 (EMD Millipore MAB8616) used in immunofluorescence (1:200); mouse mAb anti-V5 (Bio-Rad MCA1360) used in immunoprecipitation (20ug/30ul beads) and western blot (1:10,000); rabbit polyclonal antibody 746-868 (Oliver et al., 2009. J. Virol 83, 7495-7506) used in western blot (1:4,000); VZV moused mixed mAb (Meridian Life Sciences C05108MA) used for immunohistochemistry at 1:2,000
Validation	All antibodies were validated for each experimental setting for optimum dilution prior to use for the acquisition of quantitative data. The specificity of antibodies were determined by a lack of cross reactivity with non-specific proteins. The specificity of human mAb 93k was demonstrated in the present study. In addition, the specificity of mouse mAb 206 and rabbit polyclonal antibody 746-868 have been demonstrated previously (Montalvo & Grose, 1986. Virology 149, 230-241; Oliver et al., 2009. J. Virol 83, 7495-7506). The specifications of commercially available antibodies can be found on the manufacture's website using their catalogue numbers.

Eukaryotic cell lines

Policy information about cell lines

Cell line source(s)	MeWo cells were originally from the ATCC(HTB-65). CHO-DSP1 and Mel-DSP2 were developed in house (Yang et al., 2016. J. Virol 90, 7567-7578).

Authentication	MeWo cells were not authenticated further. The CHO-DSP-1 and Mel-DSP2 were authenticated in experimental assays using postive controls that demonstrate the reconstitution of the split luciferase-green fluorescent proteins DSP1 and DSP2 to yield luciferase and GFP activity.
Mycoplasma contamination	Cell lines have not recently been tested for mycoplasma contamination.
Commonly misidentified lines (See ICLAC register)	Commonly misidentified cells lines were not used.

Flow Cytometry

Plots

Confirm that:

- [x] The axis labels state the marker and fluorochrome used (e.g. CD4-FITC).
- [x] The axis scales are clearly visible. Include numbers along axes only for bottom left plot of group (a 'group' is an analysis of identical markers).
- [x] All plots are contour plots with outliers or pseudocolor plots.
- [x] A numerical value for number of cells or percentage (with statistics) is provided.

Methodology

Sample preparation	CHO-DSP1 cells transfected with VZV glycoprotein B mutants or glycoprotein H control.
Instrument	FACSCalibur flow cytometer.
Software	FlowJo CE
Cell population abundance	Only a single cell type was present, CHO-DSP1, and 20,000 counts per sample were collected.
Gating strategy	FSC and SSC were used to gate on single cell populations for the transfected CHO-DSP1. Positive values were determined from cells transfected with VZV glycoprotein H as a negative control

- [x] Tick this box to confirm that a figure exemplifying the gating strategy is provided in the Supplementary Information.
