# Supplementary material for: A glycoprotein B-neutralizing antibody structure at 2.8 Å uncovers a critical domain for herpesvirus fusion initiation
Source: Nat Commun. 2020 Aug 18;11:4141. doi: 10.1038/s41467-020-17911-0 (PMC7435202; doi:10.1038/s41467-020-17911-0)
Supplement: Supplementary file 4 — Description of Additional Supplementary Files [file 41467_2020_17911_MOESM4_ESM.pdf]

Description for Additional Supplementary Files

File name: Supplementary Data 1

Description: Molecular interactions calculated using Find Contacts (Chimera).

This spreadsheet provides all of the contacts at the interface between VZV gB and mAb 93k calculated using the Find Contacts (Chimera).

File name: Supplementary Data 2

Description: Statistical analyses of plaque sizes for the VZV gB DIV mutants.

This spreadsheet provides the details for statistical analyses performed on the plaque sizes of VZV gB DIV mutants compared to WT pOka.

File name: Supplementary Movie 1
Description: The 2.8Å cryo-EM map and model of native VZV gB in complex with the neutralizing human mAb 93k. The movie shows the cryo-EM map of the gB-93k complex with gB colored gray and 93k colored blue. One protomer for gB is segmented into its five domains and each domain colored cyan (DI), green (DII), yellow (DIII), orange (DIV), red (DV) and pink (linker regions). The variable heavy (VH) and light (VL) chains of mAb 93k bound to this segmented protomer are colored blue (VH) and dodger blue (VL). Each domain separates and the structure models are shown for each domain to demonstrate the fit of the amino acid side chains into the cryo-EM map.

File name: Supplementary Movie 2
Description: Cryo-EM map and model at the gB-93k interface. The movie focusses on the interface of the gB-93k complex and highlights the four regions at gB β23, 25-26 (orange), gB β28-30 (orange), 93k VL CDR, CDR2 (dodger blue) and 93k VH CDR1, CDR3 (blue). Each of the four regions are shown individually to demonstrate the amino acid side chain fitment of the model into the cryo-EM map. The structure models for each region are then colored according to the Q-score values as for Suppl. Fig. 3.

File name: Supplementary Movie 3
Description: Molecular interactions at the VZV gB-93k interface. The interactions between the gB and mAb 93k amino acids identified by Find Contacts (Chimera) are highlighted in the movie. A segmentation of a single gB protomer with the 93k Fab are shown that transitions to a segmentation and model of the gB-93k interface as for Figure 2A to C. The side chains of amino acids that interact between gB and 93k are colored green with dashed lines (magenta) representing the molecular interactions between the labelled amino acids; gB (orange), 93k VH (blue) and 93k VL (dodger blue). Oxygen and nitrogen are colored red and blue in the amino acid side chains.

File name: Supplementary Movie 4
Description: Surface electrostatic potential of the mAb 93k Fab VH and VL chains. The movie performs a 'flyby' of the gB-93k interface where the electrostatic potential of the VH and VL chains of mAb 93k are provided (calculated using APBS; Adaptive Poisson-Boltzmann Solver [1]

with the gB interface residues represented in stick format (orange). The potentials are on a red–white–green color map (-2.5 to 2.5) in units of kJ/mol/e.

Reference in the legend for Supplementary Movie 4.

1 Jurrus, E. *et al.* Improvements to the APBS biomolecular solvation software suite. *Protein Sci* 27, 112-128, doi:10.1002/pro.3280 (2018).
