# Supplementary material for: A glycoprotein B-neutralizing antibody structure at 2.8 Å uncovers a critical domain for herpesvirus fusion initiation
Source: Nat Commun. 2020 Aug 18;11:4141. doi: 10.1038/s41467-020-17911-0 (PMC7435202; doi:10.1038/s41467-020-17911-0)
Supplement: Supplementary file 2 — Peer Review File [file 41467_2020_17911_MOESM2_ESM.pdf]

Reviewer #1 (Remarks to the Author):

Olivier and colleagues present in this work the structure, by cryo-EM at 2.8Å resolution, of varicella-zoster virus (VZV) native gB protein in complex with a neutralizing human monoclonal antibody, 93k. The glycoprotein gB together with gH-gL forms the viral fusion complex and post-fusion structures of gB ectodomain have been solved for several members of the Herpesviridae family to which VZV belongs. However, the structure of native pre-fusion gB remains elusive. In this paper, the authors isolate native gB from VZV infected cells and use a neutralizing antibody to identify protein domains important for viral fusion. Using several mutants, designed based on the structural data, they identify domain IV, that is distant from the gB fusion loop located in DI, as a critical region required for herpesvirus fusion.

The paper is well written and the quality of the figures and especially the movies is remarkable. I enjoyed reading this work and I have two main points that I feel should be addressed more explicitly in the text concerning the conformation of gB and the mechanism of action of the 93k antibody.
The neutralizing 93k antibody presumably acts by binding to the prefusion form of gB and the approach to isolate native gB from VZV-infected cells would suggest the possibility to capture gB in the pre-fusion conformation. Why the recovered native protein is in post-fusion conformation? Was any pre-fusion/antibody complex recovered?
What is the hypothesized mechanism of action of 93k? If 93k blocks fusion should not prevent the prefusion to postfusion transition? Is it possible that the MOA intervenes at levels other than fusion (receptor? binding of other viral proteins?).

The native full length gB protein has been isolated from the infected cells but the structure reported is the one of the ectodomain. Western blot analysis with an antibody recognizing the cytoplasmic region of the protein showed that the protein in the complex included the transmembrane domain. Was this part of the complex visible in the cryo-EM structure (as it seems from figure 1D)?

Minor points concerning the figures and the presented results are following:

Figure 1. This figure is too dense and especially the upper part depicting the functional assays is too small. The foci and syncytia are barely visible to be appreciated. The two bar graphs in 1A and 1B could be fused showing UT – 93k – 93kF -SG2 – 206 data in one graph for each figure. For figure 1A the y axis of the graph should indicate PFU or N. of plaques and not PFU/ml. It would be easier to express the values as percent of untreated. I assume that the two bands in the WB of figure 1C are the results of the furin cleavage of gB but this is not mentioned in the text. I would add a color legend indicating DI – DII etc in figure 1E. I would also remove the bottom part of figure 1F which is well detailed in suppl. figure 3 as well as in figure 2. This will allow more space to enlarge the top of the figure.

Figure 4. Figure 4B is too small. I would also show the error bars in black otherwise it seems that the top of the empty bars is filled and it may induce some confusion in the understanding of the graph. In my opinion, the authors underestimate the impact of the difference in expression (especially at the cell surface) of the mutant proteins in the cell-cell fusion assay. It is possible that a defined threshold is required for cell-cell fusion to occur so even small variation in protein expression may have a higher impact in the fusion read-out. The fusion data for each mutant should be normalized for the amount of surface protein expressed. Also, the authors could comment on the different signal with the SG2 and 93k antibodies, I would have expected some loss of signal with 93k because the mutants are targeting the antibody binding site but why some mutants seem to react less with SG2 than 93k? This would imply that the binding domain of 93k is overlapping with the one of SG2 (as suggested later in the paper) and the mutation is affecting the recognition of this antibody too. In this case to quantify expression of the mutants should not another antibody (not affected by the mutations) be used? Could the authors comment on the following observations? Figure 4E: The 596-AA-597 mutant does not show the cleaved fragments when immunoprecipitated with 93k but these forms are present in the

immunoprecipitation with SG2. Figure 4F: the western blot for gB shows only one band, is the cleaved form not part of the gB/gH-gL complex?
The control gH lane indicated in the legend (pag.44 line881) is not depicted in the figure.

Figure 5. The E670A mutant seems to drastically affect fusion both in cell-cell fusion assay and in the context of the infectious virus. Why the double mutant (which include this mutation) behaves differently in the context of the virus infection? Figure 5F: If the gH-V5 protein was used for the coimmunoprecipitation assay, why gH-WT line does not show a band with the V5 antibody?

Suppl. Figure 2. The naming of the figures is shifted B should be A, C should be B etc.

Reviewer #2 (Remarks to the Author):

Members of the Herpesviridae are pathogens for human and animal diseases. The glycoprotein gB mediates essential physiological process like viral entry and membrane fusion. However the mechanism for gB pre-post fusion transition and the factors triggering membrane fusion are still unclear. This study reported a neutralizing human mAb 93k targeting VZV gB domain IV (DIV) and found the 93k inhibits membrane fusion mediated by VZV gB. The authors further determined the 2.8Å cryo-EM structure of native full-length VZV gB trimer in complex with mAb 93k Fab fragments. Based on the high resolution cryo-EM structures, the authors further used mutation and functional assays to identify critical residues from the 93K epitopes on the DIV for gB-mediated membrane fusion initiation. The authors also analyzed these key positions among other Herpesviridae gB orthologues by sequence alignment and structural comparison, thereby extending the insights from the current VZV study to other herpesviruses. I think this study is of high quality, both in structure determination and the subsequent mutation and functional assays.

Here are my questions and comments:

1. The Figure 3C clearly shows the interacting residues between the DIV beta stands and the antibody 93K. Besides the important residues in the beta23 and beta30 revealed as in Figures 4 and 5, the authors did not check the residues of other beta stands, especially the beta25. Will these interacting residues in other beta stands also have potential influence on fusion?

2. The authors mentioned other reported DIV antibodies of gB orthologues (HCMV antibody and HSV-1 SS10) and the SS10 have key residues corresponding beta30 of VZV gB. I advise the authors to show their epitope patch in the structure for clear comparison. The SS10-interacting resides in the beta30 of HSV-1 gB are also important for fusion of HSV-1?

3. Will the antibody 93k affect the interaction between VZV gB and heparan sulfate proteoglycans?

4. There are no control and gB-WT images in Figure 5C and 5D, respectively.

5. In Figure 4F and 5F, the WB-93 should be WB-93K.

6. In Figure 2, Figure 3B and Figure 3C, the images of interacting residues are all too crowded. Especially in Figure 3C different panels, there is no need to show residues not related to interact with specific DIV residues.

<u>Author's responses to comments from reviewers #1 and #2.</u>

REVIEWER #1

Olivier and colleagues present in this work the structure, by cryo-EM at 2.8Å resolution, of varicella-zoster virus (VZV) native gB protein in complex with a neutralizing human monoclonal antibody, 93k. The glycoprotein gB together with gH-gL forms the viral fusion complex and post-fusion structures of gB ectodomain have been solved for several members of the Herpesviridae family to which VZV belongs. However, the structure of native pre-fusion gB remains elusive. In this paper, the authors isolate native gB from VZV infected cells and use a neutralizing antibody to identify protein domains important for viral fusion. Using several mutants, designed based on the structural data, they identify domain IV, that is distant from the gB fusion loop located in DI, as a critical region required for herpesvirus fusion.

The paper is well written and the quality of the figures and especially the movies is remarkable. I enjoyed reading this work and I have two main points that I feel should be addressed more explicitly in the text concerning the conformation of gB and the mechanism of action of the 93k antibody.

The neutralizing 93k antibody presumably acts by binding to the prefusion form of gB and the approach to isolate native gB from VZV-infected cells would suggest the possibility to capture gB in the pre-fusion conformation. Why the recovered native protein is in post-fusion conformation? Was any pre-fusion/antibody complex recovered?

<u>Reply</u>: Unfortunately, a prefusion gB-antibody complex was not identified with our current purification protocol. The cryo-EM data were mined for such complexes, but class averages were not identified that resembled a prefusion form of gB in complex with mAb 93k Fab fragments. The prefusion form of herpesvirus gB has been challenging to purify for herpesviruses in general. Currently, the only evidence of prefusion forms of herpesvirus gB have been in the context of purified virions or transiently expressed gB on the surface of exosomes[1,2]. In addition, specialized approaches used for other viral fusogens such as pH manipulation or mutagenesis to 'lock' the fusion protein has not currently been feasible for herpesvirus gB.

What is the hypothesized mechanism of action of 93k? If 93k blocks fusion should not prevent the prefusion to postfusion transition? Is it possible that the MOA intervenes at levels other than fusion (receptor? binding of other viral proteins?).

<u>Reply</u>: This is a fascinating question and one we would really like to resolve. However, the precise MOA behind the fusion inhibition of mAb 93k remains elusive. We agree that there are three potential interventions for mAb 93k; prevention of gB to bind with other viral proteins, inhibition of binding to a cell surface protein, or locking the gB in a prefusion conformation. Currently, we predict that mAb 93k does not block binding to other viral proteins. This is supported by our minimal gB/gH-gL fusion assay and immunoprecipitation experiments (Fig. 1B and Suppl. Fig. 7). As gH is required to prime gB for fusion, mAb 93k would need to prevent this

interaction. However, mAb 93k inhibits fusion but can also co-immunoprecipitate gH. Thus, the gB-gH protein-protein interaction remains intact, but fusion is prevented. The second MOA, inhibition of binding to a cell surface protein, remains an open question for VZV because a bona fide gB-cell surface protein has not been identified to date. This is likely due to the complex interplay between gB/gH-gL and the subsequent interactions required with cell surface proteins needed to trigger the fusion reaction. For some herpesviruses, gH-gL plays a role in cell tropism by binding cell surface proteins[3-7] *enabling the priming of gB. Due to the minimal fusion requirement for VZV, only requiring gB, gH and gL, our current hypothesis is that gH-gL interacts with cell surface proteins. Thus, it remains unclear if mAb 93k inhibits fusion via interfering with interactions between gB and cell surface proteins. The final MOA, preventing a transition from a prefusion to a postfusion state, is currently the most plausible based on the data provided in the current manuscript. However, to prove such a MOA the structure would have to be resolved in a similar fashion as that of RSV*[8] *where gB would need to be co-expressed in the presence of mAb 93k Fab fragments. Such approaches have been attempted with HSV gB but with mixed results and only low resolution structures*[9]*. It is an approach that we are considering for future structural biology projects.*

The native full length gB protein has been isolated from the infected cells but the structure reported is the one of the ectodomain. Western blot analysis with an antibody recognizing the cytoplasmic region of the protein showed that the protein in the complex included the transmembrane domain. Was this part of the complex visible in the cryo-EM structure (as it seems from figure 1D)?

<u>Reply</u>: *Both the transmembrane domain and CTD were indeed visible in the gB-93k complex. However, owing to their flexibility these domains were not resolved to a high resolution. When the display threshold in the cryo-EM map is lowered, the domains become visible but high-resolution structural details are not resolvable. Focused classification of these domains was applied but resolvable structures continued to prove elusive. Stable alpha-helical structures were not evident in the VZV gB CTD in the cryo-EM class averages used for map reconstruction. This contrasts with HSV-1 gB, where it has been possible to partially resolve the structure for the CTD using X-ray crystallography*[10]*. The reasons behind the lack of structure for the VZV CTD are unclear but for HSV it was likely possible due to the crystal packing of the protein, which has the possibility to stabilize the flexible structures and induce the formation of ordered structures. This was evident in the HSV-1 gB structure as the CTD helices of one trimer were shaped by DIV of the second trimer in the crystal packing as the gB trimers formed a head-to-tail configuration*[10]*; biochemical studies provided some support to the presence of helices in the HSV-1 gB CTD*[11] *but these have not been confirmed for VZV.*

Minor points concerning the figures and the presented results are following:

Figure 1. This figure is too dense and especially the upper part depicting the functional assays is too small.

<u>Reply</u>: *We have broken up Fig. 1 into two separate figures to improve visibility.*

The foci and syncytia are barely visible to be appreciated. The two bar graphs in 1A and 1B could be fused showing UT – 93k – 93kF -SG2 – 206 data in one graph for each figure. For figure 1A the y axis of the graph should indicate PFU or N. of plaques and not PFU/ml. It would be easier to express the values as percent of untreated.

Reply: We prefer to use PFU rather than applying a normalization to the data for plaque assays. This is a convention that we have adhered to in our previously published studies.

I assume that the two bands in the WB of figure 1C are the results of the furin cleavage of gB but this is not mentioned in the text.

Reply: Thanks for picking this up. A note about furin cleavage has been added to the figure legend.

I would add a color legend indicating DI – DII etc in figure 1E.

Reply: Thanks for the suggestion. A legend has been added to the renumbered Fig. 2B.

I would also remove the bottom part of figure 1F which is well detailed in suppl. figure 3 as well as in figure 2. This will allow more space to enlarge the top of the figure.

Reply: We have addressed the issue of space by generating two figures from the original Fig. 1. The lower portion of 1F (now 2D) provides an overview of gB DIV and mAb 93k VH and VL chains and exposure of the internal portions of the gB-93k interface in an exploded view. In addition, the 2D static scenes were captured from Suppl. Movie 2. Suppl. Fig. 3 provides additional quantitative analysis of the exploded interface and are also captured scenes from Suppl. Movie 2.

Figure 4. Figure 4B is too small.

Reply: We have increased the size of the original Figure 4B, now 5B, to improve visibility.

I would also show the error bars in black otherwise it seems that the top of the empty bars is filled and it may induce some confusion in the understanding of the graph.

Reply: With the increase in the size of Fig. 5B (previously 4B) the error bars are now distinguishable for each column.

In my opinion, the authors underestimate the impact of the difference in expression (especially at the cell surface) of the mutant proteins in the cell-cell fusion assay. It is possible that a defined threshold is required for cell-cell fusion to occur so even small variation in protein expression may have a higher impact in the fusion read-out. The fusion data for each mutant should be normalized for the amount of surface protein expressed.

Reply: We agree that there are occasions where surface levels of glycoproteins affect fusion. However, in our experience and from our published studies, the correlation between cell surface levels of gB and fusion is not linear and fusion can occur despite relatively low levels of surface expression of gB mutants compared to WT gB. We address this point in detail in the next paragraph. As the fusion data are already normalized to WT we feel that it's not appropriate to perform a second normalization to the quantities of gB detected by mAbs 93k or SG2 on the

surface of the cells. Importantly, and as noted by the reviewer in the next comment, the mutations target the epitope, so it is not clear whether there is less gB on the cell surface or that the antibodies have reduced binding to gB. We have added a sentence in the results to clarify this point; lines 150 to 155.

Also, the authors could comment on the different signal with the SG2 and 93k antibodies, I would have expected some loss of signal with 93k because the mutants are targeting the antibody binding site but why some mutants seem to react less with SG2 than 93k?

Reply: *Based on our hypothesis outlined above, the mutations of gB are likely to affect fusion function and reduce the ability of gB to undergo a conformational change from a prefusion to a postfusion state. We know that the mAb 93k epitope is accessible in both the prefusion and post fusion forms of gB. However, the accessibility of the SG2 epitope in the prefusion form is currently unknown. As SG2 does not neutralize well or inhibit fusion, these data imply that the SG2 epitope might be partially masked in the prefusion form of gB. Unlike mAb 93k, reduced fusion function does correlate with the inability of mAb SG2 to detect gB on the cell surface (see data provided in Fig. 1 below), but only for the beta23 mutants. A correlation was not seen between cell surface expression and fusion function for the beta30 mutants, something we have noted in our previous publications that target other locations in gB and also with gH[12-15]. Although our data support the notion that the mutations affect the ability of gB to enact a wild type-like conformational change, without structural or further biochemical data this remains a hypothesis.*

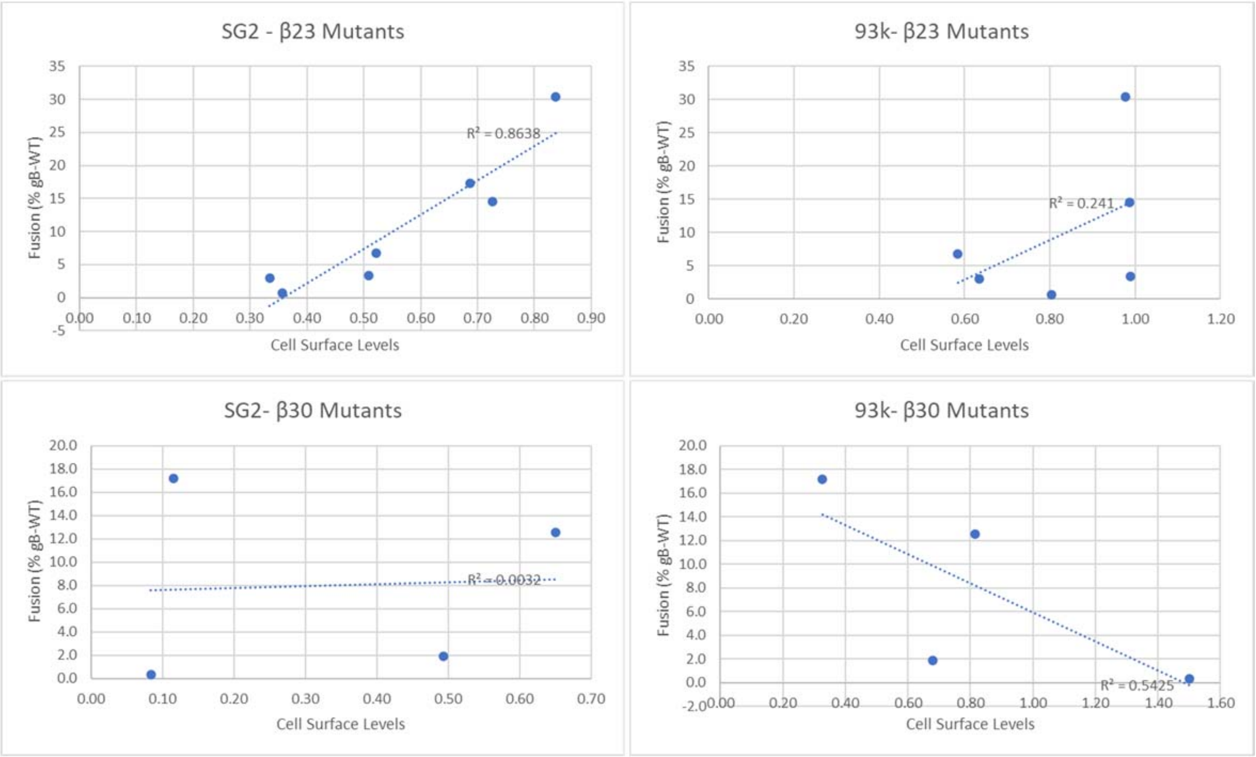

Fig. 1. Correlation of cell-cell fusion with surface levels of VZV gB mutants. The total amount of gB detected by either mAbs SG2 or 93k was used to normalize surface levels.

This would imply that the binding domain of 93k is overlapping with the one of SG2 (as suggested later in the paper) and the mutation is affecting the recognition of this antibody too. In this case to quantify expression of the mutants should not another antibody (not affected by the mutations) be used?

Reply: *We agree with the implication that the epitopes of mAbs SG2 and 93k have some level of overlap. We have a manuscript in preparation demonstrating some but not complete overlap of the epitopes. The SG2 mAb has been used in our previous studies to demonstrate cell surface levels of gB*[12,15]*. Unfortunately, mAbs SG2 and 93k are the only antibodies available for VZV gB. Our rabbit polyclonal antibody cannot be used to detect cell surface gB because the epitope is only exposed on the cytoplasmic domain*[16]*. As the purpose of these experiments was to demonstrate that gB could be detected on the cell surface we were left with relying on combined data from mAbs SG2 and 93k. Although we recognize there is a differential between the two antibodies, these data demonstrate that gB is trafficked to the cell surface at levels we have previously demonstrated to induce cell-cell fusion.*

Could the authors comment on the following observations? Figure 4E: The 596-AA-597 mutant does not show the cleaved fragments when immunoprecipitated with 93k but these forms are present in the immunoprecipitation with SG2. Figure 4F: the western blot for gB shows only one band, is the cleaved form not part of the gB/gH-gL complex?

Reply: *It is unclear to us why the furin cleavage product is absent for the mAb 93k immunoprecipitation of gB mutant* ^{596}*AA*^{597}*. As this mutant is defective in both fusion and producing viable VZV, and we predict that the epitopes of mAbs 93k and SG2 are differentially accessible in prefusion and postfusion conformations, we surmise that the* ^{596}*AA*^{597} *prevents a necessary conformational change of gB. In turn, this prevents canonical cleavage of gB by furin, which can only be immunoprecipitated by mAb 93k in the prefusion conformation. The SG2 mAb on the other hand will only immunoprecipitate gB that has undergone furin cleavage, potentially exposing the mAb SG2 epitope.*

The control gH lane indicated in the legend (pag.44 line881) is not depicted in the figure.

Reply: *The text has been edited accordingly; "The gH control lane where CHO cells were transfected with gH-WT is shown in Fig. 6E." page 47, lines 908-909.*

Figure 5. The E670A mutant seems to drastically affect fusion both in cell-cell fusion assay and in the context of the infectious virus. Why the double mutant (which include this mutation) behaves differently in the context of the virus infection?

Reply: *This was a surprising observation and the reason why we perform mutations in the VZV genome in addition to biochemical assays. We predict that the E670A substitution alters the chemical environment in relation to Y667. The loss of negative charge might enable the hydroxyl group of Y667 to form aberrant interactions either within gB or with other VZV proteins inactivating the virus. In contrast, the Y667A and the double* ^{667}*A/A*^{670} *would not suffer the same effect because the hydroxyl group is absent. There might be other chemistries that could explain this phenomenon, but these are beyond the scope of the manuscript.*

Figure 5F: If the gH-V5 protein was used for the coimmunoprecipitation assay, why gH-WT line does not show a band with the V5 antibody?

<u>Reply</u>: The gH-WT was used as a negative control and does not contain a V5 tag.

Suppl. Figure 2. The naming of the figures is shifted B should be A, C should be B etc.

<u>Reply</u>: Thanks for spotting this. Corrected.

REVIEWER #2

Members of the Herpesviridae are pathogens for human and animal diseases. The glycoprotein gB mediates essential physiological process like viral entry and membrane fusion. However the mechanism for gB pre-post fusion transition and the factors triggering membrane fusion are still unclear. This study reported a neutralizing human mAb 93k targeting VZV gB domain IV (DIV) and found the 93k inhibits membrane fusion mediated by VZV gB. The authors further determined the 2.8Å cryo-EM structure of native full-length VZV gB trimer in complex with mAb 93k Fab fragments. Based on the high resolution cryo-EM structures, the authors further used mutation and functional assays to identify critical residues from the 93K epitopes on the DIV for gB-mediated membrane fusion initiation. The authors also analyzed these key positions among other Herpesviridae gB orthologues by sequence alignment and structural comparison, thereby extending the insights from the current VZV study to other

herpesviruses. I think this study is of high quality, both in structure determination and the subsequent mutation and functional assays.

Here are my questions and comments:

1. The Figure 3C clearly shows the interacting residues between the DIV beta stands and the antibody 93K. Besides the important residues in the beta23 and beta30 revealed as in Figures 4 and 5, the authors did not check the residues of other beta stands, especially the beta25. Will these interacting residues in other beta stands also have potential influence on fusion?

<u>Reply</u>: This is an interesting question and one that we had also considered. The beta25 residues could potentially influence fusion. However, the mAb 93k interactions with these residues is minimal and akin to those for S589, which we used as a control in our studies. This implies that the residues in beta25 are less likely to influence fusion compared to the residues in beta23 and beta30. In addition, the mutagenesis in beta23 and beta30 that disrupt mAb 93k binding also suggest that the beta25 residues are not a critical factor in mAb 93k neutralization and fusion inhibition.

2. The authors mentioned other reported DIV antibodies of gB orthologues (HCMV antibody and HSV-1 SS10) and the SS10 have key residues corresponding beta30 of VZV gB. I advise the

authors to show their epitope patch in the structure for clear comparison. The SS10-interacting resides in the beta30 of HSV-1 gB are also important for fusion of HSV-1?

<u>Reply</u>: We have added an additional figure (Fig. 7) to show the footprint of mAb 93k on VZV gB. Our study is the first to directly target herpesvirus gB DIV for point mutagenesis. It is at this moment unclear what effects the related mutations in HSV-1 gB will have on fusion, but we predict that they will be similar. The SS10-interacting epitope was not precisely mapped but correspond to a coarse mapping performed by truncation mutagenesis[17]. This region was found to overlap with the equivalent of VZV beta30 in HSV-1 gB.

3. Will the antibody 93k affect the interaction between VZV gB and heparan sulfate proteoglycans?

<u>Reply</u>: Although heparan sulphate proteoglycans (HSPG) have been demonstrated to have roles in herpesvirus infection, it is not known whether VZV gB binds to HSPG, although this is plausible given that gB homologues for other herpesviruses do bind HSPGs. However, based on studies with HSV-1, mAb 93k is unlikely to affect binding to HSPG. Deletion of gB residues ^{68}KPKKNKKPK^{76} from HSV-1 gB reduces HSPG binding by 80%, but only in the presence of a gC deletion[18]. This partially implicates the ^{68}KPKKNKKPK^{76} region of gB in HSPG binding but also demonstrated that additional glycoproteins can bind to HSPG. In addition, the ^{68}KPKKNKKPK^{76} region of HSV-1 gB correlates to residues 75-83 in VZV gB, which is outside of the mAb 93k binding site.

4. There are no control and gB-WT images in Figure 5C and 5D, respectively.

<u>Reply</u>: The control images for 5C and 5D were omitted for space saving purposes. The controls were the same as those in Fig 4C and 4D (now 5C and 5D). These data were from the same set of experiments. We have edited the figure legend (Fig.6 previously Fig. 5) and made reference to the controls in Fig. 5C and 5D (previously Fig. 4).

5. In Figure 4F and 5F, the WB-93 should be WB-93K.

<u>Reply</u>: Thanks for spotting this. WB-93 has been changed to WB-93k in both figures; now 5F and 6F.

6. In Figure 2, Figure 3B and Figure 3C, the images of interacting residues are all too crowded. Especially in Figure 3C different panels, there is no need to show residues not related to interact with specific DIV residues.

<u>Reply</u>: As the figures represent snap shots of scenes from Suppl. Movie 3, we would like to keep these for consistency between the different viewing formats an as an aid for readers to comprehend the orientation of the gB monomer. <u>Note</u>: Fig.2 and Fig. 3 are now Fig. 3 and Fig. 4.

<u>References.</u>

1 Si, Z. *et al.* Different functional states of fusion protein gB revealed on human cytomegalovirus by cryo electron tomography with Volta phase plate. *PLoS Pathog* **14**, e1007452, doi:10.1371/journal.ppat.1007452 (2018).

2 Zeev-Ben-Mordehai, T. *et al.* Two distinct trimeric conformations of natively membrane-anchored full-length herpes simplex virus 1 glycoprotein B. *Proc Natl Acad Sci U S A* **113**, 4176-4181, doi:10.1073/pnas.1523234113 (2016).
3 Chen, J., Zhang, X., Schaller, S., Jardetzky, T. S. & Longnecker, R. Ephrin Receptor A4 is a New Kaposi's Sarcoma-Associated Herpesvirus Virus Entry Receptor. *mBio* **10**, doi:10.1128/mBio.02892-18 (2019).
4 Grosskopf, A. K. *et al.* A conserved Eph family receptor-binding motif on the gH/gL complex of Kaposi's sarcoma-associated herpesvirus and rhesus monkey rhadinovirus. *PLoS Pathog* **14**, e1006912, doi:10.1371/journal.ppat.1006912 (2018).
5 Martinez-Martin, N. *et al.* An Unbiased Screen for Human Cytomegalovirus Identifies Neuropilin-2 as a Central Viral Receptor. *Cell* **174**, 1158-1171 e1119, doi:10.1016/j.cell.2018.06.028 (2018).
6 Sathiyamoorthy, K. *et al.* Structural basis for Epstein-Barr virus host cell tropism mediated by gp42 and gHgL entry glycoproteins. *Nat Commun* **7**, 13557, doi:10.1038/ncomms13557 (2016).
7 Wu, Y. *et al.* Human cytomegalovirus glycoprotein complex gH/gL/gO uses PDGFR-alpha as a key for entry. *PLoS Pathog* **13**, e1006281, doi:10.1371/journal.ppat.1006281 (2017).
8 McLellan, J. S. *et al.* Structure of RSV fusion glycoprotein trimer bound to a prefusion-specific neutralizing antibody. *Science* **340**, 1113-1117, doi:10.1126/science.1234914 (2013).
9 Fontana, J. *et al.* The Fusion Loops of the Initial Prefusion Conformation of Herpes Simplex Virus 1 Fusion Protein Point Toward the Membrane. *mBio* **8**, doi:10.1128/mBio.01268-17 (2017).
10 Cooper, R. S., Georgieva, E. R., Borbat, P. P., Freed, J. H. & Heldwein, E. E. Structural basis for membrane anchoring and fusion regulation of the herpes simplex virus fusogen gB. *Nat Struct Mol Biol* **25**, 416-424, doi:10.1038/s41594-018-0060-6 (2018).
11 Silverman, J. L., Greene, N. G., King, D. S. & Heldwein, E. E. Membrane requirement for folding of the herpes simplex virus 1 gB cytodomain suggests a unique mechanism of fusion regulation. *J Virol* **86**, 8171-8184, doi:10.1128/JVI.00932-12 (2012).
12 Oliver, S. L. *et al.* An immunoreceptor tyrosine-based inhibition motif in varicella-zoster virus glycoprotein B regulates cell fusion and skin pathogenesis. *Proc Natl Acad Sci U S A* **110**, 1911-1916, doi:10.1073/pnas.1216985110 (2013).
13 Vleck, S. E. *et al.* Structure-function analysis of varicella-zoster virus glycoprotein H identifies domain-specific roles for fusion and skin tropism. *Proc Natl Acad Sci U S A* **108**, 18412-18417, doi:10.1073/pnas.1111333108 (2011).
14 Yang, E., Arvin, A. M. & Oliver, S. L. The cytoplasmic domain of varicella-zoster virus glycoprotein H regulates syncytia formation and skin pathogenesis. *PLoS Pathog* **10**, e1004173, doi:10.1371/journal.ppat.1004173 (2014).
15 Yang, E., Arvin, A. M. & Oliver, S. L. The Glycoprotein B Cytoplasmic Domain Lysine Cluster Is Critical for Varicella-Zoster Virus Cell-Cell Fusion Regulation and Infection. *J Virol* **91**, doi:10.1128/JVI.01707-16 (2017).
16 Oliver, S. L. *et al.* Mutagenesis of varicella-zoster virus glycoprotein B: putative fusion loop residues are essential for viral replication, and the furin cleavage motif contributes to pathogenesis in skin tissue in vivo. *J Virol* **83**, 7495-7506, doi:10.1128/JVI.00400-09 (2009).
17 Bender, F. C. *et al.* Antigenic and mutational analyses of herpes simplex virus glycoprotein B reveal four functional regions. *J Virol* **81**, 3827-3841, doi:10.1128/JVI.02710-06 (2007).
18 Laquerre, S. *et al.* Heparan sulfate proteoglycan binding by herpes simplex virus type 1 glycoproteins B and C, which differ in their contributions to virus attachment, penetration, and cell-to-cell spread. *J Virol* **72**, 6119-6130 (1998).

REVIEWERS' COMMENTS:

Reviewer #1 (Remarks to the Author):

All my comments have been throughly addressed by the authors. I just have an additional suggestion for the point below.

My first comment:
The neutralizing 93k antibody presumably acts by binding to the prefusion form of gB and the approach to isolate native gB from VZV-infected cells would suggest the possibility to capture gB in the pre-fusion conformation. Why the recovered native protein is in post-fusion conformation? Was any pre-fusion/antibody complex recovered?

Authors' reply: Unfortunately, a prefusion gB-antibody complex was not identified with our current purification protocol. The cryo-EM data were mined for such complexes, but class averages were not identified that resembled a prefusion form of gB in complex with mAb 93k Fab fragments. The prefusion form of herpesvirus gB has been challenging to purify for herpesviruses in general. Currently, the only evidence of prefusion forms of herpesvirus gB have been in the context of purified virions or transiently expressed gB on the surface of exosomes1,2. In addition, specialized approaches used for other viral fusogens such as pH manipulation or mutagenesis to 'lock' the fusion protein has not currently been feasible for herpesvirus gB.

My suggestion: I think it would be worth to mention this in the paper. It would be expected to have a prefusion native gB protein in cells yet all the purified form is in post-fusion even when the mAb 93k antibody is added shortly after the first step of purification. This underscores the highly metastable status of the prefusion form (confirmed by the absence of even minor classes resembling a prefusion form in cryo-EM data) and would suggests alternative approaches to capture the prefusion form. I wonder if a cell-permeable nanobody of mAb93k would be helpful in this task.

Reviewer #2 (Remarks to the Author):

The raised questions have been approximately addressed by the authors.

<u>Author's responses to comments from reviewers #1 and #2.</u>

REVIEWER #1

All my comments have been throughly addressed by the authors. I just have an additional suggestion for the point below.

My first comment:

The neutralizing 93k antibody presumably acts by binding to the prefusion form of gB and the approach to isolate native gB from VZV-infected cells would suggest the possibility to capture gB in the pre-fusion conformation. Why the recovered native protein is in post-fusion conformation? Was any pre-fusion/antibody complex recovered?

Authors' reply: Unfortunately, a prefusion gB-antibody complex was not identified with our current purification protocol. The cryo-EM data were mined for such complexes, but class averages were not identified that resembled a prefusion form of gB in complex with mAb 93k Fab fragments. The prefusion form of herpesvirus gB has been challenging to purify for herpesviruses in general. Currently, the only evidence of prefusion forms of herpesvirus gB have been in the context of purified virions or transiently expressed gB on the surface of exosomes1,2. In addition, specialized approaches used for other viral fusogens such as pH manipulation or mutagenesis to 'lock' the fusion protein has not currently been feasible for herpesvirus gB.

My suggestion: I think it would be worth to mention this in the paper. It would be expected to have a prefusion native gB protein in cells yet all the purified form is in post-fusion even when the mAb 93k antibody is added shortly after the first step of purification. This underscores the highly metastable status of the prefusion form (confirmed by the absence of even minor classes resembling a prefusion form in cryo-EM data) and would suggests alternative approaches to capture the prefusion form. I wonder if a cell-permeable nanobody of mAb93k would be helpful in this task.

<u>Reply</u>: Thank you for your suggestions about the additional text and future alternative approaches using a cell-permeable nanobody. We have added two sentences in the results section at lines 104-108; 'Since gB was purified from VZV infected MeWo cells, it was anticipated that alternative conformations of gB would be isolated, including prefusion forms. However, class averages were not identified in the cryo-EM micrographs that resembled a prefusion form of gB in complex with mAb 93k Fab fragments, underscoring the highly metastable status of the prefusion form of herpesvirus gB.'

REVIEWER #2

The raised questions have been approximately addressed by the authors.
